# Phase I study of local radiation and tremelimumab in patients with inoperable locally recurrent or metastatic breast cancer

**DOI:** 10.18632/oncotarget.26893

**Published:** 2019-04-26

**Authors:** Di (Maria) Jiang, Anthony Fyles, Linh T. Nguyen, Benjamin G. Neel, Adrian Sacher, Robert Rottapel, Ben X. Wang, Pamela S. Ohashi, Srikala S. Sridhar

**Affiliations:** ^1^ Department of Medical Oncology, Princess Margaret Cancer Centre, University Health Network, University of Toronto, Toronto, Canada; ^2^ Department of Radiation Oncology, Princess Margaret Cancer Centre, University Health Network, University of Toronto, Toronto, Canada; ^3^ Ontario Cancer Institute, Princess Margaret Cancer Centre, University Health Network, Toronto, Canada; ^4^ Department of Immunology, Faculty University of Toronto, Toronto, Canada; ^5^ Department of Medical Biophysics, University of Toronto, Toronto, Canada; ^6^ Laura and Isaac Perlmutter Cancer Center, NYU Langone Health, New York, NY, USA

**Keywords:** radiation, immunotherapy, breast cancer, anti-CTLA-4

## Abstract

Immunotherapy has shown modest activity in metastatic breast cancer (MBC). In this phase I dose escalation study, we assessed safety of tremelimumab, a humanized anti-CTLA4 monoclonal antibody, at starting dose 3 mg/kg, on the third day of palliative radiotherapy (2000cGy in 5 daily fractions) in patients with MBC. Primary objective was to determine the maximum tolerated dose (MTD) of tremelimumab combined with RT. Secondary objective was to assess response. Among 6 patients enrolled between July 2010 and October 2011, 5 had hormone receptor-positive MBC, 1 had triple negative MBC. Median age was 45 years. Common toxicities included lymphopenia (83%), fatigue (50%) and rash (33%). One dose-limiting toxicity occurred at 6 mg/kg, however the trial closed before MTD could be determined. One patient discontinued treatment due to a pathological fracture. Best response was stable disease (SD), 1 patient had SD for >6 months. Median follow up was 27.0 months. Median OS was 50.8 months, with 1 patient surviving >8 years. Peripheral blood mononuclear cell (PBMC) profiles showed increasing proliferating (Ki67+) Treg cells 1 week post treatment in 5 patients. Overall, tremelimumab at 3 mg/kg combined with RT appears to be a tolerable treatment strategy. Further studies are needed to optimize this combination approach.

## INTRODUCTION

Breast cancer is the most commonly diagnosed cancer and the second leading cause of cancer death in women [1]. Conventional treatment options for metastatic breast cancer (MBC) include endocrine therapy, targeted therapy and cytotoxic chemotherapy, all of which have demonstrated limited treatment response and duration. More recently, there has been increasing recognition of the key role of the host immune response in tumor response. Immune checkpoint inhibitors (ICI) have found increasing use since the first FDA approval of ipilumumab for metastatic melanoma in 2011. Multiple ICIs have now been developed and approved in other disease sites. Tremelimumab is a fully humanized monoclonal antibody targeting the checkpoint receptor CTLA-4.

Despite the remarkable activity of ICI in some settings, most patients fail to respond. In triple negative breast cancer (TNBC), overall response rates (ORR) for pembrolizumab, avelumab, and atezolizumab are in the range of 2.8%–26%, although durable responses have been reported [2–5]. Preclinical studies suggest that ionizing radiation (IR) can enhance the systemic antitumor immune response through multiple mechanisms. IR leads to cell death and subsequent release of tumor antigens as well as upregulation of MHC class I molecules, which may augment the activation of tumor-specific cytotoxic T cell and NK cell responses [6–9]. IR induced cytoplasmic double-stranded DNA is sensed by the cyclic GMP-AMP synthase (cGAS)-stimulator of interferon genes (STING) pathway to induce IFN-I, a key mediator of dendritic cell recruitment and maturation [10]. In the tumor microenvironment, proinflammatory chemotactic factors induced by IR also enhance recruitment of effector T cells [11, 12] and antigen presenting cells [7, 13–15]. In preclinical models of breast cancer, tumors unresponsive to anti-CTLA4 antibody are sensitized following IR [16]. These preclinical studies provide a strong rationale for combining radiotherapy (RT) and ICI to overcome immunotherapy resistance in MBC. We therefore performed an investigator-initiated phase I study to further evaluate the safety of this combination strategy in human subjects with MBC.

## RESULTS

### Patient characteristics

From July 2010 to October 2011, 6 female patients were enrolled. Their characteristics are summarized in Table 1. Median age was 45 years. None had significant comorbidities at study entry. Five patients had hormone receptor-positive MBC, 1 had recurrent metastatic triple negative breast cancer (TNBC) with an original diagnosis of hormone receptor-positive breast cancer. HER2-positive disease was seen in 1 patient; 2 patients had unknown HER2 status (their pathology specimens were obtained externally in 1997 and 2001 and were unavailable for review). Two patients presented with *de novo* metastatic disease; 4 had recurrent metastatic disease, with a median time to relapse of 9.0 years.

**Table 1:** Baseline characteristics at study enrollment

Clinicopathologic features	*n* = 6
Median age	45.0 [range 42.6, 60.2]
Family history for breast or ovarian cancer	
No	4
Yes	2
*De novo* metastatic disease	
No	4
Median time to metastatic relapse^*^	9.0 years [range 2.9, 12.6]
Yes	2
ECOG performance status at enrollment	
0	0
1	6
2	0
3	0
4	0
Histology	
Ductal	6
Lobular	0
Histopathology grade	
1	0
2	2
3	2
Unknown	2
ER/PR	
Positive	5
Negative	1
HER2	
Positive	1
Negative	3
Unknown	2
Number of metastatic sites	
1	0
2	2
3	3
4	1
Presence of visceral metastases	5
Previous radiotherapy	
No	2
Yes	4
Previous endocrine therapy	
No	4
Yes	2
Previous lines of palliative chemotherapy	
0	1
1	0
2	3
≥3	2

^*^Calculated from date of surgery for locally advanced breast cancer to date of diagnosis of metastatic relapse

Most patients had visceral organ involvement and received prior chemotherapy such as taxane, capecitabine, epirubicin, platinum and cyclophosphamide. One patient received sunitinib plus herceptin as part of a clinical trial prior to enrollment. Two patients previously received endocrine therapy (tamoxifen). The number of subsequent therapies ranged from 0-6, and included endocrine and chemotherapy regimens, such as vinorelbine, gemcitabine, capecitabine and cyclophosphamide.

### Dose escalation and determination of MTD

Three subjects received tremelimumab at dose level 1 (3 mg/kg). As no DLTs occurred at this level, the trial escalated to the next dose level. Among the 3 patients dosed at level 2 (6 mg/kg), 1 developed grade 3 confusion and presyncope, which constituted a DLT. According to the study protocol, cohort expansion with another 3 patients at dose level 2 would be required to determine MTD. Unfortunately, this trial was not able to meet target accrual. One patient (subject 4) received a subsequent dose of tremelimumab at dose level 2 at the investigator’s discretion for ongoing stable disease (SD), 90 days after the first dose.

### Toxicities

Toxicities are shown in Table 2. Four patients (67%) developed grade >3 toxicity, which included fatigue, pathological fracture, confusion/presyncope and asymptomatic lymphopenia. Two of these 4 patients were dosed at level 1 (pathological fracture, lymphopenia, and fatigue); the other 2 were dosed at level 2 (confusion and presyncope, lymphopenia). There were no grade 4 or 5 adverse events. One patient discontinued the study due to a pathologic fracture 7.1 weeks after receiving tremelimumab, requiring urgent surgery. One patient experienced grade 3 confusion and presyncope (DLT)approximately 3 weeks following therapy, which required intravenous hydration in hospital.

**Table 2:** Adverse events

Adverse events	Grade 1 *n*	Grade 2 *n*	Grade 3 *n*	Grade 4 *n*
Diarrhea	1	0	0	0
Rash	2	0	0	0
Fatigue	2	0	1	0
Pathologic fracture from radiated site	0	0	1	0
Dyspnea	1	0	0	0
Anemia	0	1	0	0
Neutropenia	0	1	0	0
Lymphopenia	1	1	3	0
Confusion, presyncope^*^	0	0	1	0

^*^dose limiting toxicity

Five of 6 patients developed grade 1-3 lymphopenia within a median of 0.9 weeks (range 0.7, 1.7) following tremelimumab, whereas neutropenia occurred in only 1 patient. There were no transaminitis, renal dysfunction or endocrinopathies following treatment. Other common toxicities included fatigue (50%) and rash (33%), occurring within 8 weeks following therapy. Grade 1 diarrhea and dyspnea each occurred in 1 patient approximately 1 week after receiving tremelimumab.

### Efficacy

As shown in Table 3, overall disease control rate was 33%; however, there were no objective responses. One patient (subject 4) had SD for more than 6 months. At data cutoff (January 1, 2019), median follow up after tremelimumab administration was 27.0 months (4.8–101.7). For the 3 patients who were alive at the last visit, median follow up was 41.6 months (37.3–101.7). Median PFS was 1.5 months, and Median OS was 50.8 months from date of MBC diagnosis, and 27.0 months from tremelimumab administration.

**Table 3:** Treatment outcomes of the study cohort

Subject #	Receptor status		Previous therapy				Trial RT			Study drug				Efficacy				Worst AE
	ER/PR	HER2	RT	Systemic therapy regimen	Surg	Site	Dose (Gy)	#	Time to trial discontinuation (mo)	Dose mg (mg/kg)	Number of doses	Reason for DC	Best OR^+^	OS1^*^ (mo)	OS2^**^ (mo)	PFS^**^ (mo)	Grade	Type
1	+	NA	Yes	None	Yes	right humerus	2000	5	2.2	195.0 (3)	1	PD	SD	103.4	94.0	2.8	1	Diarrhea, rash, fatigue
2	+	NA	Yes	Tamoxifen Docetaxel, capecitabine	Yes	T4	2000	5	1.4	140.7 (3)	1	SAE	PD	53.9	36.9	1.7	3	T4 pathologic fracture, lymphopenia
3	+	+	No	Sutent + herceptin (trial), docetaxel + herceptin, adrimaycin, taxol + HKI-272 (trial), tamoxifen	No	T spine	2000	5	1.5	239. 1 (3)	1	PD	PD	53.9	16.7	1.6	3	Fatigue
4	+	-	No	Docetaxel Abraxane	Yes	right ribs	2000	5	6.1	385.2 (6), 360.6 (6)	2	PD	SD	47.7	41.6	6.1	2	Neutropenia
5	-^^^	-	Yes	Cisplatin + gemcitabine, Cyclophosphamide+ veliparib (trial)	Yes	left chest nodule	2000	5	1.3	357.6 (6)	1	PD	PD	9.7	4.8	1.5	3	Lymphopenia
6	+	-	Yes	Tamoxifen capecitabine	No	left femur	2000	5	0.9	414.6 (6)	1	PD	PD	32.5	16.2	0.9	3	Lymphopenia, confusion and presyncope; grade 2 anemia

^*^calculated from date of metastatic breast cancer diagnosis

^**^calculated from date of first dose of tremelimumab initiation

^+^According to RECIST v1.0 criteria

^^^At time of original breast cancer diagnosis, receptor status was ER positive, PR positive and HER2 negative

ER: estrogen receptor; PR: progesterone receptor; HER2: human epidermal growth factor receptor 2; NA: not available; RT: radiotherapy; Surg: surgery; DC: discontinue; OR: overall response PD: progressive disease; SAE: severe adverse events; SD: stable disease

Subject 1 survived more than 8 years (101 months) after receiving tremelimumab. Following disease progression (PD), this patient received endocrine therapy (letrozole for 6 years then tamoxifen for more than 2 years) and remains on tamoxifen at the last follow up. Unlike other subjects, subject 1 did not receive any palliative systemic therapy prior to enrollment.

### Correlative immune profiling

**Figure 1:**
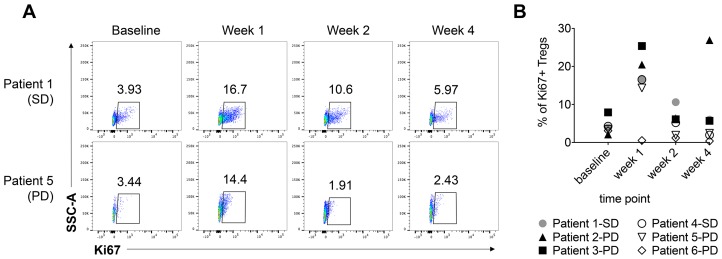
The percentage of Ki67+ regulatory T cells increases at 1 week post-radiation therapy and tremelimumab treatment. The percentage of Ki67+ Tregs (CD3+CD4+FOXP3+CD25+CD127-) in PBMCs isolated from serial blood samples was analyzed by flow cytometry. (**A**) Representative Ki67 staining in the Treg compartment at baseline, week 1, week 2, and week 4 is presented for two patients, and the percentage of Ki67+ cells is indicated for each time point. (**B**) The percentage of Ki67+ Tregs at the indicated timepoints is shown for all six patients. The best response for each patient is indicated in the figure legend. SD = stable disease; PD = progressive disease.

Immune profiling by flow cytometry was performed on prospectively collected peripheral blood mononuclear cells. No consistent changes in CD4^+^ or CD8^+^ T cell populations were seen across the baseline, week 1, week 2, week 3 and week 4 samples. CD28 expression remained largely stable on CD8^+^ T cells, however in patient 6, CD28 expression decreased from 92% at baseline to 62% at week 4. The percentage of Ki67+ proliferating regulatory T cells (Tregs: CD3+CD4+FOXP3+ CD25+ CD127–) seemed to increase at week 1 for patients 1–5 (from <12% to up to 36%) then decreased close to baseline in most patients (Figure 1). Overall, there were no notable trends in our flow cytometric analyses that were associated with SD versus PD.

Neutrophil to lymphocyte ratio (NLR) was also explored. Interestingly, 3 subjects (subject 2, 5 and 6) had relatively higher pretreatment NLR (>4) and developed significant elevation of NLR (>10) within 3 weeks following tremelimumab therapy, and all 3 patients developed PD. The 2 patients who achieved SD had lower pretreatment NLRs (1.8 in subject 1; 1.1 in subject 4) than others (2.6 to 6.3), and maintained NLR <2 through week 4 following tremelimumab.

## DISCUSSION

In 2012, Postow et al first described a patient whose metastatic melanoma regressed upon treatment with ipilimumab and concurrent palliative RT of 28.5 Gy [17]. Since this publication, other similar series have emerged [18–20]. To our knowledge, the only phase III study using this combination strategy with published results is CA184-043. This trial demonstrated that ipilimumab (anti-CTLA4 antibody) plus to a conservative dose of palliative bone-directed RT (8 Gy × 1), which did not improve overall survival (OS) in heavily pre-treated metastatic prostate cancer patients, however treatment was tolerable [21].

With the ongoing interest in this combination strategy, the safety profile of ICI plus higher dose RT requires systematic and prospective evaluation. Although our study was unable to meet target accrual to determine the MTD, tremelimumab at 3 mg/kg did not induce any DLTs and therefore appears safe and feasible to combine with RT. Existing studies also report acceptable safety profile [22, 23], with grade 3 or 4 toxicity [21, 24, 25] similar to ICI monotherapy [26]. Follow up of prior studies was generally 6 months or less [22, 23]. Our longer follow up provides additional assurance that this combination approach is feasible.

The most common toxicity was lymphopenia, all occurring within 2 weeks following tremelimumab. In 3 of the 5 patients, lymphopenia was grade 3. Lymphocytes are radiosensitive [27]. Interestingly, subject 1 did not develop lymphopenia and had prolonged OS, with ongoing disease stability with endocrine therapy 8 years post-progression from study treatment. Subject 4, who had only grade 1 lymphopenia, had the longest duration of disease control. All other patients with grade >1 lymphopenia had PD within 1.5 months of therapy. Severe radiation-induced lymphopenia [28–30], high NLR pre- and post-treatment [31, 32] are known to correlate with poor prognosis. Lymphopenia and high NLR may be markers of poor T cell reserve and therefore associate with lower response to ICI. Other toxicities included a T4 pathological fracture at 7 weeks in subject 2, highlighting the need for special caution in treating large spinal metastases with this combination approach, especially when considering stereotactic body radiation therapy. No new safety signals were detected otherwise.

The best ORR was SD, and no abscopal effects were observed. One patient had SD over 6 months, which suggests durable responses are possible with this combination strategy. Although our sample size was too small to discern any definitive association, ICI may be more effective in earlier treatment settings. Notably, subject 1 with the exceptionally long OS had no prior therapies at enrollment, unlike other subjects. Subject 4 had more prolonged disease control, and received only 2 lines of prior therapy. As chemotherapy suppresses the immune system, patients who are less pre-treated likely have a more intact repertoire of immune cells which are sensitive to activation by ICI. Post ICI therapy, chemotherapy may produce superior antitumor activity via an increased number of antigen-specific CD8+ T cells within the tumor microenvironment [33, 34]. Retrospective studies suggest chemotherapy has a higher response rate pre-ICI relative to the post-ICI setting [35, 36]. In the Keynote-086 trial, pembrolizumab showed a much higher ORR in TNBC patients who were previously untreated (21.4%, cohort B), compared with those who received prior chemotherapy (5.3%, cohort A) [37, 38]. Similar findings of favorable responses in treatment-naïve patients were also seen with atezolizumab [3]. Interestingly, although the CA184-043 trial did not demonstrate improved OS overall, there was a significant benefit in patients with low burden, non-visceral metastatic disease, suggesting RT plus ICI may be more effective in less advanced disease [21]. Further research is warranted to elucidate the optimal treatment setting of this combination strategy.

Following PD on study, 3 subjects were alive at the last follow up. Subject 1 survived at leastmore than 8 years on endocrine therapies, subject 2 and 3 had ≥ 5 lines of subsequent therapies and survived for at leastmore than 48 months after since diagnosis of MBC. This is compared to a median of 3 lines of palliative therapy [39] and median OS of 27-37 months in other contemporary series [39, 40]. This could reflectThere could be some selection bias for younger patients with excellent performance status and more favorable disease biology, as evident from the exceptionally long median time to recurrence in those with recurrent MBC. However, despite short PFS on study treatment, it is unknown whether the combination strategy of ICI plusand RT contributed to the long median OS by augmenting disease activity or response post progression.

We did not identify any major trends in the correlative analysis of PBMCs, however patients 1–5 exhibited a notable increase in the percentage of proliferating (Ki67+) Tregs at week 1 post-treatment. Although PBMCs may not be representative of the immune response in the tumor, preclinical experiments have found more robust Treg proliferation in comparison to other T cell subsets in the tumor microenvironment [41]. Tregs are more radio-resistant than other T cells [42], and increasing Treg populations in peripheral blood following chemotherapy and RT has been observed [43, 44]. The radio-resistance of Tregs may preferentially select for survival of these cells post-radiotherapy, which could result in an increased proportion of proliferating Tregs at week 1. These Tregs are able to not only survive the effect of IR, but also retain proliferative potential given the expression of Ki67. Whether the increased percentage of proliferating Tregs at week 1 reflects other factors such as activation of immunosuppression pathways by RT, and the non-immunogenic phenotype of the tumor itself, remains unclear. Interestingly, the percentage and number of reconstituting Tregs in the peripheral blood have been negatively associated with treatment response [45, 46]. Consistent with the increased proportion of Ki67+ cells in the Treg compartment post-treatment, the overall frequency of Tregs increased post-treatment in patients 1, 3, and 4; but not by more than two-fold. There is data to suggest that hyper-activation of Tregs can result in Treg-specific apoptosis in the tumor microenvironment [47]. It is possible then that these Ki67+ Tregs go on to die, but we cannot make this conclusion based on our immune profiling data alone. In patients 1–5, where we observed an increase in the percentage of Ki67+ Tregs, the proportion of Tregs in the peripheral blood does not decrease below baseline levels by week 4. Furthermore, higher tumor-infiltrating CD8/Treg ratios have been linked with better outcomes in some breast cancers [48, 49]. We observed less than two-fold increases in the proportion of Tregs in patients 1, 3 and 4, and accordingly, less than two-fold decreases in the CD8/Treg ratio (as percentages of CD3+ T cells). The proliferation and frequency of Tregs within the tumor microenvironment – in addition to the tumor-infiltrating CD8/Treg ratio – after treatment with radiation and tremelimumab, would be an interesting biomarker to evaluate in future studies.

Future investigations are also needed to define the optimal strategy of combining RT and immunotherapy. Current evidence suggests fractionated RT [8, 50, 51] and RT doses >10Gy, such as those used in our study, achieve enhanced systemic antitumor immune responses [17, 52, 53]. Concurrent ICI and RT is favored over sequential administration [54, 55], and multiple doses of ICI are likely required to mount a meaningful systemic immune response [56]. In our study, most patients received only 1 dose of tremelimumab, which may be insufficient to produce synergy. Proton radiation, which spares surrounding normal tissue better than photon therapy, and hypofractionation of RT (used in this study) may better preserve peripheral lymphocytes and synergize better with ICI [57, 58]. Irradiation of pathologic draining LNs compared with other sites might also improve cross-presentation of tumor associated epitopes by dendritic cells [8].

This trial had some important limitations. Patient accrual was challenging and limited our ability to determine MTD and draw meaningful conclusions. This deficiency highlights the need for cross-disciplinary collaboration in such combination trials. At the time of trial initiation, immune-oncology was still at its infancy. Investigators lacked knowledge of irRECIST criteria and treatment beyond progression. Our patient population was not selected to enrich response. We now know that the tumor mutational load might correlate with ICI response [59–62], and unselected MBC typically displays lower numbers of somatic mutations, and less engagement with T cells [63]. However, certain breast cancer subtypes have higher degree of immune infiltration and PD-L1 expression, such as TNBC and HER2-positive MBC [64–69]. In a phase II study of TNBC using a similar treatment protocol with pembrolizumab given within 3 days of RT delivered over 5 daily fractions, ORR reached 33%, and ongoing responses were still present after 20 weeks [56].

Taken together, our results with relatively long follow up demonstrate safety profile which support testing ICI in combination with RT in future prospective trials.

## MATERIALS AND METHODS

### Study design and patients

In this investigator-initiated, open-label phase 1 dose escalation trial, we enrolled women aged 18 years or older with incurable, histologically confirmed MBC requiring palliative radiation therapy (RT). Patients were enrolled between July 2010 and October 2011 at the Princess Margaret Cancer Centre. Other inclusion criteria included Eastern Cooperative Oncology Group (ECOG) performance status of 0 or 1 and adequate organ function. There were no restrictions on previous lines of therapy (≥4 weeks must have elapsed since the last dose of systemic therapy). Patients were excluded if they had contraindications to RT, previous treatment with any anti-CTLA4 agent, history of chronic inflammatory, gastrointestinal or autoimmune disorder, insulin-dependent diabetes, active diarrhea at baseline, planned radiation to pelvic masses (to minimize the risk of colitis), history of congestive heart failure, stroke, myocardial infarction or thromboembolic event, untreated brain metastases, or concurrent or planned immunosuppressive dose of corticosteroid therapy or other immunosuppressive medication (e,g., methotrexate, rapamycin) for longer than 10 days within 4 weeks prior to enrollment or while on trial. For further information regarding the trial protocol, please see Appendix.

This study was approved by the Research Ethics Board at University Health Network, and was conducted in accordance with the Declaration of Helsinki and Good Clinical Practice Guidelines. All patients provided written informed consent before enrollment. Tremelimumab was provided by Pfizer Canada Inc. The study protocol, accrual process, data collection and analysis were proposed and conducted independently by study investigators.

### Treatment procedures

All patients received palliative external RT of 2000cGy in 5 daily fractions at one tumor site using 6-18 MV photons. The rationale for choosing this radiation dose was: 1) ability to induce sufficient cell kill in the majority of tumors and provide appropriate signals for enhancing the anti-tumor T cell response [8, 50]; and 2) 2000cGy is a standard dose for palliative radiation with low risk of toxicities [70]. On the third day of RT, tremelimumab was administered intravenously at 3, 6, 10, or 15 mg/kg, as per dose escalation rules outlined below. We chose the third day of RT to dose tremelimumab given that 1) the effect of radiation on promoting tumor antigen cross-presentation and MHC upregulation can occur as early as one day after radiation [6], and 2) subsequent CTLA4 upregulation on activated effector T cells is detectable 2 days after an antigenic signal [71]. The starting dose of tremelimumab was 3mg/kg, a dose shown to be safe as a single agent in previous trials [72, 73]. Given the long half-life of tremelimumab (22 days) [74], patients with a clinical response (or clinical benefit at the investigator’s discretion) following the first tremelimumab dose were eligible to receive a subsequent dose at 90 days, for a maximum of 4 cycles in total. Combination of subsequent cycles with radiation were not permitted.

### Evaluation of endpoints

The primary objective of this study was to assess safety and define the maximum tolerated dose (MTD) of tremelimumab in combination with radiation therapy. The MTD was the dose at which no subject experienced a life-threatening adverse event, and at which 0-1 out of 6 patients (<33%) experienced a dose limiting toxicity (DLT) within 12 weeks of tremelimumab administration. DLT was defined as any of the following: 1) any Grade 4 toxicity, 2) other grade >3 toxicities that do not recover to ≤ Grade 1 or baseline within 7 days of maximal management (including skin toxicities), or 3) other Grade >2 or greater treatment-related autoimmune toxicity of critical organs (lung, heart, kidney, bowel, bone marrow and nervous system, eye except anterior uveitis). Dose escalation rules followed a classic 3 + 3 design, with assessment of DLT at 12 weeks. If no patient experienced a DLT, the trial would proceed to the next dose level cohort. If a DLT was encountered in one of three patients, 3 additional patients would be treated at the same dose for a total of 6 patients. If one of the six patients experienced a DLT, the trial would escalate to the next dose level. If DLT was encountered in more than one patient, the MTD would be declared as having been exceeded, and dose escalation was discontinued.

Patients were evaluated for toxicity with clinical visits, bloodwork and urine analysis weekly in the first month, and every two weeks following treatment. Thyroid function tests, autoantibody panel and human antihuman antibody (HAHA) were assessed at 4 and 12 weeks. Adverse events and other symptoms were graded according to the National Cancer Institute Common Terminology Criteria for Adverse Events (CTCAE) version 3.0, as per trial protocol.

Our secondary objective was to explore clinical efficacy. ORR was assessed 6-8 weeks following tremelimumab dosing, defined according to RECIST v1.0, as per trial protocol [75]. Complete response (CR) was defined as disappearance of all target and non-target lesions. Partial response (PR) was defined as at least 30% decrease in the sum of the tumor measurements (TM) of target lesions compared to baseline. Non-target lesions could persist, provided there was no unequivocal progression in these lesions. CR and PR were confirmed by repeat assessments performed within 4–6 weeks. Progressive disease (PD) was defined as >20% increase in the sum TM of the target lesions from the smallest sum TM recorded since baseline or the appearance of one or more new lesions or unequivocal progression of existing non-target lesions. If the changes in sizes of the target and nontarget lesions did not qualify as either PR or PD, then the patient would be deemed to have stable disease (SD). Routine assessment of response with CT imaging was performed 8 weeks following tremelimumab, and every 6–8 weeks thereafter. Overall survival was defined from the date of metastatic breast cancer diagnosis (OS1) and from date of first dose of tremelimumab initiation (OS2).

Peripheral blood mononuclear cells (PBMCs) were collected at baseline, and at 1, 2, and 4 weeks following tremelimumab dosing. Cryopreserved PBMCs were thawed and stained for flow cytometric analysis. Staining was performed using antibodies purchased from BD Biosciences (CD3 (clone UCHT1), CD4 (RPA-T4)), Thermo Fisher Scientific (FOXP3 (clone 236A/E7), CD25 (BC96), CD127 (eBioRDR5), Ki67 (20Raj1), TCRγδ (B1.1), CD28 (CD28.2), and CD19 (HIB19)), and BioLegend (CD56 (clone HCD56). Data were acquired using a 5-laser LSR Fortessa X-20 (BD) and analyzed using FlowJo v.10 (Treestar).

## SUPPLEMENTARY MATERIALS




