# Supplementary material for: Phase I study of local radiation and tremelimumab in patients with inoperable locally recurrent or metastatic breast cancer
Source: Oncotarget. 2019 Apr 26;10(31):2947–58. doi: 10.18632/oncotarget.26893 (PMC6508206; doi:10.18632/oncotarget.26893)
Supplement: Supplementary file 1 [file oncotarget-10-2947-s001.pdf]

Phase I study of local radiation and tremelimumab in patients with inoperable locally recurrent or metastatic breast cancer

SUPPLEMENTARY MATERIALS

Supplementary Appendix: See Supplementary Trial protocol
