# Supplementary material for: Phase I study of local radiation and tremelimumab in patients with inoperable locally recurrent or metastatic breast cancer
Source: Oncotarget. 2019 Apr 26;10(31):2947–58. doi: 10.18632/oncotarget.26893 (PMC6508206; doi:10.18632/oncotarget.26893)
Supplement: Supplementary file 2 [file oncotarget-10-2947-s002.doc]

**CLINICAL PROTOCOL FOR AN INVESTIGATOR-INITIATED RESEARCH STUDY**

**PHASE I STUDY OF LOCAL RADIATION AND CP-675,206 ADMINISTRATION IN PATIENTS WITH INOPERABLE LOCALLY RECURRENT OR METASTATIC BREAST CANCER**

**Compound: CP-675,206 (Pfizer)**

**Version date: October 21, 2008**

**Principal Investigator/Sponsor: Srikala Sridhar, MD, MSc, FRCP(C)**

**Institution: University Health Network, Toronto, Canada**

**Co-Investigators: Anthony Fyles, MD, FRCP(C)**

**Linh Nguyen, PhD**

**Benjamin Neel, MD, PhD**

**Robert Rottapel, MA, MD, FRCP(C)**

**Pamela Ohashi, PhD**

**TABLE OF CONTENTS**

[1. SUMMARY 4](#__RefHeading___Toc181688691)

[2. INTRODUCTION 4](#__RefHeading___Toc181688692)

[2.1. Background 4](#__RefHeading___Toc181688693)

[2.1.1. Metastatic breast cancer 4](#__RefHeading___Toc181688694)

[2.2. CP-675,206 5](#__RefHeading___Toc181688695)

[2.3. CP-675,206 – Clinical Experience 6](#__RefHeading___Toc181688696)

[2.3.1. Summary 6](#__RefHeading___Toc181688697)

[2.3.2. Single-agent, Single Dose Study (A3671001) 6](#__RefHeading___Toc181688698)

[2.3.3. Phase I-II, Single agent, Multiple-Dose Study (A3671002) 7](#__RefHeading___Toc181688699)

[2.4. Rationale 10](#__RefHeading___Toc181688700)

[2.4.1. Rationale for combining local radiation with CTLA-4 blockade 10](#__RefHeading___Toc181688701)

[2.4.2. Rationale for the radiation therapy dose 11](#__RefHeading___Toc181688702)

[2.4.3. Rationale for the timing of CP-675,206 administration 11](#__RefHeading___Toc181688703)

[2.5. Future directions 11](#__RefHeading___Toc181688704)

[2.6. Timeline 13](#__RefHeading___Toc181688705)

[3. TRIAL OBJECTIVES 13](#__RefHeading___Toc181688706)

[3.1. Primary Objective 13](#__RefHeading___Toc181688707)

[3.2. Secondary Objectives 13](#__RefHeading___Toc181688708)

[4. TRIAL DESIGN 13](#__RefHeading___Toc181688709)

[4.1. Overview 13](#__RefHeading___Toc181688710)

[4.2. Dose Escalation Rules 14](#__RefHeading___Toc181688711)

[4.3. MTD Evaluable Patient - Definition 14](#__RefHeading___Toc181688712)

[4.4. Dose Limiting Toxicities 15](#__RefHeading___Toc181688713)

[4.5. Maximum Tolerated Dose 15](#__RefHeading___Toc181688714)

[5. STUDY POPULATION 15](#__RefHeading___Toc181688715)

[5.1. Inclusion criteria 15](#__RefHeading___Toc181688716)

[5.2. Exclusion criteria 16](#__RefHeading___Toc181688717)

[5.3. Enrollment criteria 17](#__RefHeading___Toc181688718)

[6. TRIAL TREATMENTS 17](#__RefHeading___Toc181688719)

[6.1. Trial Period 17](#__RefHeading___Toc181688720)

[6.1.1. Dose Levels 18](#__RefHeading___Toc181688721)

[6.1.2. Patient Dose Modifications (CP-675,206) 18](#__RefHeading___Toc181688722)

[6.1.3. Patient Dose Modifications (Radiation Therapy) 18](#__RefHeading___Toc181688723)

[6.1.4. Management of CP-675,206 Toxicity 18](#__RefHeading___Toc181688724)

[6.1.5. Management of Hypersensitivity Reactions 21](#__RefHeading___Toc181688725)

[6.2. CP-675,206 Drug supply 22](#__RefHeading___Toc181688726)

[6.2.1. CP-675,206 - Preparation and Dispensing 22](#__RefHeading___Toc181688727)

[6.2.2. Administration 22](#__RefHeading___Toc181688728)

[6.3. Concurrent Medication(s) 22](#__RefHeading___Toc181688729)

[6.4. Salvage Therapy 23](#__RefHeading___Toc181688730)

[6.5 Additional cycles 23](#__RefHeading___Toc181688731)

[7. TRIAL PROCEDURES 23](#__RefHeading___Toc181688732)

[7.1. Pretrial Assessments 23](#__RefHeading___Toc181688733)

[7.2. On-Trial Assessments 25](#__RefHeading___Toc181688734)

[7.3. Follow-up Visits 27](#__RefHeading___Toc181688735)

[7.3.1. Treatment-Related Adverse Event Follow-Up 27](#__RefHeading___Toc181688736)

[7.3.2. Disease Progression Follow-Up 28](#__RefHeading___Toc181688737)

[7.3.3. Survival Follow-Up 28](#__RefHeading___Toc181688738)

[7.4. Patient Withdrawal 28](#__RefHeading___Toc181688739)

[8. STUDY ASSESSMENTS 29](#__RefHeading___Toc181688740)

[8.1. Safety Assessments 29](#__RefHeading___Toc181688741)

[8.2. Clinical response assessments 29](#__RefHeading___Toc181688742)

[8.2.1. Measurability of Tumor Lesions 29](#__RefHeading___Toc181688743)

[8.2.2. “Target” and “Non-Target” Lesions 30](#__RefHeading___Toc181688744)

[8.2.3. Techniques for Assessing Measurable Disease 31](#__RefHeading___Toc181688745)

[8.2.4. Objective Response Classifications 31](#__RefHeading___Toc181688746)

[8.2.5. Frequency of Tumor Measurements 32](#__RefHeading___Toc181688747)

[8.2.6. Evaluation of Overall Response 32](#__RefHeading___Toc181688748)

[8.3. Immunological monitoring 33](#__RefHeading___Toc181688749)

[9. ADVERSE EVENT REPORTING 33](#__RefHeading___Toc181688750)

[9.2. Reporting requirements 33](#__RefHeading___Toc181688751)

[10. DATA ANALYSIS/STATISTICAL CONSIDERATIONS 34](#__RefHeading___Toc181688752)

[10.1. Trial Populations 34](#__RefHeading___Toc181688753)

[10.2. Sample Size Determination 34](#__RefHeading___Toc181688754)

[10.3. Dose Escalation 35](#__RefHeading___Toc181688755)

[10.4. Analysis of Primary Endpoint 36](#__RefHeading___Toc181688756)

[10.4.1. Safety Analysis 36](#__RefHeading___Toc181688757)

[10.5. Analysis of Secondary Endpoints 36](#__RefHeading___Toc181688758)

[10.5.1. Efficacy Analyses 36](#__RefHeading___Toc181688759)

[10.5.2. Analysis of Other Endpoints 37](#__RefHeading___Toc181688760)

[10.5.3. Interim Analysis 37](#__RefHeading___Toc181688761)

[11. DATA HANDLING AND RECORD KEEPING 37](#__RefHeading___Toc181688762)

[11.1. Retention of Patient Records and Study Files 37](#__RefHeading___Toc181688763)

[12. ETHICS 38](#__RefHeading___Toc181688764)

[12.1. Research Ethics Board Approval for Protocols 38](#__RefHeading___Toc181688765)

[12.2. Informed Consent 38](#__RefHeading___Toc181688766)

[13. TABLES **Error! Bookmark not defined.**](#__RefHeading___Toc181688767)

[Table 1. Pre-trial assessments **Error! Bookmark not defined.**](#__RefHeading___Toc181688768)

[Table 2. On-trial assessments 41](#__RefHeading___Toc181688769)

[14. REFERENCES 43](#__RefHeading___Toc181688770)

# 1. SUMMARY

Patients with inoperable locally recurrent or metastatic breast cancer requiring local radiotherapy will be given local radiation to one tumor site and systemic CP-675,206. Successive cohorts of subjects will be given escalating doses of CP-675,206 in order to determine the maximum tolerated dose of CP-675,206 when given in combination with radiation. The primary endpoint will be toxicity. The secondary endpoints will be clinical and immunological responses. This trial is being conducted as a prelude to a planned phase II trial of CP-675,206 in combination with radiation in metastatic breast cancer.

# 2. INTRODUCTION

## **2.1. Background**

### **2.1.1. Metastatic breast cancer**

Breast cancer is the most common cancer in Canadian women. In 2007 an estimated 22,300 women will be diagnosed and 5300 will die of the disease. Despite advances in therapy, metastatic disease remains an incurable illness, with a median survival of only 2 years. Standard systemic treatment options for metastatic disease include chemotherapy (taxanes, anthracyclines, capecitabine, vinorelbine, gemcitabine) or hormonal therapy (tamoxifen, aromatase inhibitors). Radiation is frequently used in the metastatic setting for palliation of symptoms, with the most frequent site of radiation being bone. Response rates to first line chemotherapy are in the range of 30%, however these responses are not durable. Currently there are no curative options for metastatic disease, underscoring the need for novel therapeutic approaches.

CTLA4 is a receptor expressed on the surface of activated T cells and regulatory T cells. Since signalling through CTLA4 negatively regulates T cell activity, blockade of CTLA4 has emerged as a potential therapeutic strategy to enhance anti-tumor T cell responses (1). CTLA-blockade has been tested in clinical trials using humanized monoclonal antibodies, and some biological responses have been reported. The anti-tumor immune response may be further augmented by the combination of CTLA4-blockade with radiation, with the potential to mediate regression of metastases outside of the field of radiation. The primary goal of this study therefore is to establish the safety of CTLA4-blockade using the antibody CP-675, 206 in combination with radiation. This trial is being conducted as a prelude to a planned phase II trial of CP-675,206 in combination with radiation in metastatic breast cancer.

## **2.2. CP-675,206**

*Additional information can be found in the CP-675,206 Investigator’s Brochure.*

CP-675,206 is a fully human monoclonal antibody (mAb). It binds to the CTLA4 molecule, which is expressed on the surface of activated T lymphocytes and T regulatory cells. The binding of CTLA4 to its target ligands (B7-1 and B7-2) provides a negative regulatory signal, which limits T-cell activation. CP-675,206 antagonizes binding of CTLA4 to B7 ligands and enhances human T-cell activation as demonstrated by increased cytokine (interleukin-2, interferon-γ) production in vitro in whole blood or peripheral blood mononuclear cell cultures. CP-675,206 is thought to stimulate patients’ immune systems to attack their tumors by blocking the negative regulatory signal of CTLA4.

There is emerging evidence that T regulatory cells may play a role in cancer progression or may present a barrier to immunotherapy (2). Increased numbers of T regulatory cells with an immune suppressive phenotype have been observed in the peripheral blood of patients with cancer, including breast cancer (3). Thus, blocking CTLA4 function on T regulatory cells may also result in enhanced immunotherapy.

In patients, CP-675,206 exhibits a biphasic PK profile following IV infusion. The mean systemic exposure parameters (maximum plasma concentrations [Cmax] and area under the plasma concentration-time curve [AUC]) increase with dose in an approximately proportional manner. Based on the data collected from the single dose phase 1 Trial A3671001, CP-675,206 has a low clearance (0.132 mL/h/kg), a small volume of distribution at steady state (81.2 mL/kg), and a long terminal half-life (22.1 days). These values are consistent with those of natural IgG2.

The anti-tumor activity of antibodies to CTLA4 has been demonstrated in a variety of murine tumor models. In 1996, enhancement of antitumor immunity by CTLA4 blockade was shown for the first time in mice. Here, in vivo administration of antibodies to CTLA4 resulted in the rejection of tumors, including pre-established tumors. In addition, this rejection resulted in immunity to a secondary exposure to tumor cells, suggesting that blockade of the inhibitory effects of CTLA4 can allow for, and potentiate, effective immune responses against tumor cells (4).

CTLA-blockade has been tested in clinical trials using humanized monoclonal antibodies, and some biological responses have been reported. For example, tumor necrosis and vitiligo have been observed using the antibody MDX-010 (5), and a 14% objective response rate was reported from a study with 198 patients with melanoma or renal cell carcinoma (6). The antibody CP-675,206 has been shown to induce durable tumor responses in patients with metastatic melanoma in Phase 1 and Phase 2 clinical studies (7, 8, 9). For instance, in a dose escalation study using CP-675,206, 39 patients, mostly with melanoma, were treated with one dose between 0.01 and 15mg/kg. Two complete responses and 2 partial responses were observed (9).

Since CTLA4-blockade is not dependent on vaccination with specific tumor antigens, this approach may also be effective in different types of cancer, including breast cancer. Furthermore, combining CTLA4-blockade with other therapies that impact anti-tumor immunity, such as radiation therapy, may enhance clinical responses.

## **2.3. CP-675,206 – Clinical Experience**

### **2.3.1. Summary**

CP-675,206 is an investigational agent, and its safety profile has not been fully established. As of August 27, 2006, the safety database is comprised of data from 454 patients treated in completed and ongoing studies.

Single-agent CP-675,206 has shown early evidence of efficacy in patients with melanoma, when administered both as a single dose and in the multiple dose setting.

### **2.3.2. Single-agent, Single Dose Study (A3671001)**

Study A3671001 enrolled a total of 44 patients. At study entry 1 patient (2.3%) had a colorectal tumor, 39 patients (88.6%) had melanoma, and 4 patients (9.1%) had urethral or kidney tumors. All patients enrolled were analyzed for AEs. Dosing: Cohorts of at least 3 patients were recruited for each dose level in the escalation sequence of CP-675,206 from 0.01 to 15 mg/kg. Study Discontinuations: All patients completed the single-dose study. There were no treatment-related AEs leading to discontinuations.

#### 2.3.2.1. Trial A3671001 - Safety

The most frequently observed treatment-emergent, all-causality AEs (in ≥10% of patients) were rash (29.5%), nausea (27.3%), diarrhea (27.3%), fatigue (25.0%), pruritus (22.7%), headache (18.2%), asthenia (13.6%), vomiting (13.6%), fever (11.4%), peripheral edema (11.4%), weight loss (11.4%), dizziness (11.4%), and anemia (11.4%). The incidence of rash, pruritus, and diarrhea appeared to increase with dose. Hyperthyroidism was possibly immune-mediated as evidenced by alteration in autoantibodies. Other possible immune-mediated events, such as skin hypopigmentation/vitiligo, psoriasis, hypophysitis, and alterations in pituitary activity, had no direct evidence of the mechanism of toxicity.

Treatment-emergent, treatment-related CTC Grade 3 events were limited to diarrhea (1 patient each at 6 and 10 mg/kg and 2 patients at 15 mg/kg), rash (1 patient at 15 mg/kg), enzymatic alteration (1 patient with elevated lipase at 10 mg/kg), and asthma (1 patient with pre-existing history at 10 mg/kg). Three CTC Grade 4 events were reported (respiratory arrest, respiratory failure, and malignant melanoma). In all cases, the cause for the CTC Grade 4 event was not the study drug.

#### 2.3.2.2. Trial A3671001 - Efficacy

When administered, as a single agent, CP-675,206 has been found to be associated with clinical activity in patients with metastatic melanoma (7, 8, 9).

In the single dose setting of trial A3671001, which treated primarily melanoma patients, 4 patients had objective responses (one complete response (CR) out of 3 patients treated at 3 mg/kg, one partial response (PR) out of 11 patients treated at 10 mg/kg, and one PR and one CR out of 6 patients treated at 15 mg/kg).

### **2.3.3. Phase I-II, Single agent, Multiple-Dose Study (A3671002)**

*Note: This information is included in this Study Protocol for completeness, although this trial will utilize CP-675,206 in a single dose.*

The single-agent, multiple-dose experience is limited to the ongoing Study A3671002. The phase 1 portion of study A3671002 was designed to assess the safety and tolerability of escalating doses of CP-675,206, to determine the pharmacokinetic-based recommended Phase 2 dose. The Phase 2 portion of the A3671002 trial (multiple dosing) randomized eligible patients to receive either CP-675,206 at 10 mg/kg every 28 days (Arm A) or 15 mg/kg every 90 days (Arm B). According to the database of August 27, 2006, 89 patients were enrolled and treated in the trial, 44 in Arm A and 45 in Arm B. Enrollment was completed in November 2005 and 2 patients remain active on-trial.

Diarrhea was the most frequent Grade 3 adverse event requiring medical intervention. Occasional biopsies of the colon taken at the time of diarrhea have indicated lymphocytic infiltration in the lamina propria or a composite picture resembling inflammatory bowel disease, and there have been diagnoses of colitis confirmed by biopsy. CT scans of the abdomen and a PET scan have shown bowel wall thickening. Although the mechanism behind the development of diarrhea is not clearly understood, there is an, as yet incompletely characterized, potential inflammatory component to the diarrhea, with a colitis that is histologically similar to inflammatory bowel disease (IBD). A suggested Diarrhea Management Guideline is provided within this protocol.

Important safety characteristics of both arms are presented below Fewer SAE’s, serious toxicity and trial discontinuations are associated with administering CP-675,206 at a dose of 15 mg/kg every 3 months.

#### 2.3.3.1. CP-675,206 (10 mg/kg Every 28 Days) – Safety Profile

Of the 44 patients treated with this dose and schedule, 7 (15.9%) were reported with Grade 3 diarrhea. Other Grade 3 toxicities included 3 patients (6.8%) with dehydration, 2 patients (4.5%) with colitis, 1 patient each (2.3%) with perforation of the colon, pancreatitis, arthralgia, arthritis, shoulder pain, rash, and skin exfoliation.

In more than 20% of patients, diarrhea (31.8%), nausea (20.5%), fatigue (22.7%), pruiritis (27.3%) and rash (approximately 41%) were reported. In 5 to 20% of patients, constipation (4.5%), dyspepsia (4.5%), vomiting (11.4%), fever (6.8%), decreased weight (4.5%), dehydration (4.5%), muscle spasms (6.8%), myalgia (9.1%), headache (4.5%), skin exfoliation (9.1%) and transient acantholytic dermatosis were reported.

To date, one patient has exhibited a hypersensitivity reaction shortly after the start of infusion exhibiting periorbital edema and non pruritic rash that were relieved by antihistamine. This patient experienced a similar reaction during rechallenge with CP-675,206 that was again relieved by antihistamine. The patient was then withdrawn from the trial.

#### 2.3.3.2. CP-675,206 (15 mg/kg, every 3 Months) – Safety Profile

The most frequent treatment-related adverse events were diarrhea, rash, pruritis, fatigue, and nausea (see below).

Details regarding the treatment-related Grade 3 and 4 events experienced by those patients treated at 15 mg/kg are presented below. There were 2 Grade 4 events, which were deemed treatment-related by the Investigator: (1) a patient with lipase increase in the absence of clinical signs or symptoms and (2) pulmonary embolism.

There has been one patient death, which the investigator attributed to study treatment. A 64-year-old patient with metastatic melanoma and history of diverticulitis started to receive IV CP-675,206, 1575 mg, once every 90 days, on October 25, 2006. The patient was admitted to hospital on November 2, 2006 for nausea, vomiting, fever and abdominal pain. A CT scan showed diverticulitis. On November 3, the patient died. At the time of his death, the investigator considered there was a reasonable possibility the event of 'death' was related to study drug CP-675,206. A copy of the autopsy report was forwarded to Pfizer, and based on the lack of any evidence of generalized inflammation on autopsy, the Sponsor does not attribute the events diverticulitis, nausea, vomiting, abdominal pain, and fever to administration of the study product CP-675,206. Nausea, vomiting, abdominal pain are considered secondary to diverticulitis and ruptured diverticulum.

Diverticulitis with ruptured diverticulum most likely represent a natural complication of the patient’s underlying diverticulosis. An updated causality assessment from the Investigator has not yet been provided.

#### 2.3.3.3. CP-675,206 (15 mg/kg, every 3 Months) – Efficacy

This regimen has been found to be associated with clinical activity in studies in which CP-675,206 was administered to patients with metastatic melanoma. In the phase II trial A3671002, the clinical efficacy associated with dosing 15 mg/kg every 3 months is similar to the efficacy of dosing 10 mg/kg monthly. 3/43 objective responses (CR and PR) were observed in the former group and 4/41 objective responses have been observed in the latter group.

## **2.4. Rationale**

### **2.4.1. Rationale for combining local radiation with CTLA-4 blockade**

Immunotherapeutic strategies, including CTLA-4 blockade, are aimed at inducing the immune response against tumor cells. This response may be augmented by combination with radiation. There are many potential mechanisms by which radiation therapy may enhance the effect of immunotherapy. For example, the direct induction of tumor cell death by radiation and the subsequent release of tumor antigens may augment the activation of tumor-specific T cells. Other effects of radiation on the tumor microenvironment may also lead to enhanced T cell priming and anti-tumor activity. Radiation has been shown to induce the upregulation of many molecules on human tumor cells, such as Fas/CD95 (10), B7.2 (11) and MHC class I (12) as well as adhesion molecules such as ICAM-1, VCAM-1 and P-selectin (13, 14, 15). In addition, pro-inflammatory cytokines are induced, such as IFN-γ (16). Novel proteins are induced upon irradiation, which, together with an increase in the intracellular generation of peptides, may lead to an expanded repertoire of responding T cells (17). Importantly, increases in T cell activation in the local milieu and local draining lymph nodes as a result of combination therapy can result in increased circulation of activated tumor-specific T cells and tumor regression at distant, non-irradiated tumor sites.

Studies in various murine cancer models have demonstrated the synergistic effect of combining immunotherapy with local radiation. A model of mammary carcinoma was used to investigate the combination of irradiation of the primary lesion with systemic CTLA-4 blockade (18). This regimen resulted in greatly delayed tumor growth, increased animal survival, and a decrease in lung metastases compared with either intervention alone. The anti-tumor response was dependent on CD8+ T cells, and CTLs specific for the tumor cells could be detected in the spleen following treatment. Many other studies combining local radiation with other immunotherapeutic strategies in mice have also demonstrated a synergistic effect of combination therapy (17, 19, 20, 21, 22, 23, 24).

The anti-tumor activity induced by these combination therapies was shown to be dependent on T cells, and in some cases even antigen-specific responses could be detected (19, 20, 23).

Thus, there is strong pre-clinical evidence that combining radiation therapy and immunotherapy (including CTLA4-blockade) may result in enhanced anti-tumor immune responses and systemic tumor regression. Therefore it is appropriate to study this combination therapy in humans, particularly in patients with incurable disease and very limited treatment options.

### **2.4.2. Rationale for the radiation therapy dose**

Subjects will receive 2000cGy at one tumor site, in 5 daily fractions. This dose induces significant tumor cell death in the majority of tumors, and should provide the appropriate signals for potentially enhancing the anti-tumor T cell response induced by CP-675,206. This is also the standard dose for palliation of metastatic tumors and is associated with a low risk of toxicities.

### **2.4.3. Rationale for the timing of CP-675,206 administration**

Subjects will receive radiation therapy in 5 daily fractions. On the third day of radiation, subjects will also be given one dose of CP-675,206 intravenously. The effect of radiation on promoting cross-presentation of tumor antigens and upregulation of MHC molecules can occur as early as one day after radiation (17). This cross-presentation will induce T cell activation and subsequent CTLA4 upregulation. CTLA4 upregulation on activated effector T cells is detectable 2 days after an antigenic signal (25). Thus, administration of CP-675,206 on the third day of radiation therapy should maximize the potential for CP-675,206 to enhance T cell responses specific for breast tumor-associated antigens. In addition, since the half-life of CP-675,206 in vivo is 22 days, it will still have the potential to synergize with any radiation-induced enhancement of anti-tumor immunity in the days and weeks following dosing. Indeed, the immune-enhancing effects of radiation have been observed at both early and late timepoints after radiation (26).

## **2.5. Future directions**

As described above, the combination of radiation therapy and immune-activating agents such as CP-675,206 has the potential to induce effective anti-tumor T cell responses leading to systemic tumor regression. The clinical efficacy of this combination therapy needs to be investigated in a phase II trial. Thus, the current trial is designed to provide safety data and determine the recommended phase II dose.

The planned study population for our phase II clinical trial will be enriched for patients with so called “triple negative” (ER- PR- HER2-) breast cancers. While considerable progress has been made in the development of targeted therapy for those with hormone receptor and HER2 positive disease, there is now considerable interest in the development of strategies to help patients with so called “triple negative” breast cancer. While these patients may only represent approximately 15% of all breast cancer patients, they have more limited treatment options and poorer clinical outcomes than patients with other breast cancers (27, 28).

Although combined radiation and immunotherapy could potentially lead to tumor regression in most types of breast cancer patients, recent data from triple negative breast cancers suggest that enhanced immunotherapy could have a particularly marked impact on these patients.

The rationale behind this hypothesis is that triple negative breast cancers and medullary breast cancers share basal-like histological features (29, 30) and gene expression profiles (30). Strikingly, medullary breast cancers have both an improved prognosis and immune cell infiltrates (31, 32, 33, 34). Supervised analysis of microarray data has been done to compare basal medullary breast cancers with basal ductal breast cancers (30). In this analysis, most of the genes overexpressed in medullary breast cancers were immune response genes; in particular, those associated with cell-mediated immune responses. Given these features of medullary breast cancers, it is possible that other breast cancers that share basal-like features, including basal-associated antigens (such as triple negative breast cancers) may be particularly amenable to immunotherapeutic strategies. The prediction is that enhancing immunotherapy by combining it with radiation therapy could result in a “conversion” of triple negative breast cancer into a disease with the favorable immune infiltrates and prognoses similar to those associated with medullary breast cancers.

Ultimately, the addition of CP-675,206 to a radiation treatment regimen could prove to be an exciting treatment option for patients with all types of breast cancer, for whom adjuvant radiation therapy is now standard of care. Therefore, we hope to rapidly move from phase I testing to other scenarios.

The Princess Margaret Hospital is the third largest comprehensive cancer center in North America with a long established history of excellence in clinical trials. The Medical Oncology division sees approximately 500 new breast cancer patients each year and Radiation Oncology sees 1200 new breast cancer patients each year. This trial will be conducted in collaboration with scientists from The Campbell Institute for Breast Cancer Research and the Ontario Cancer Institute at PMH, who have expertise in basic and translational immunology. The dedication of the multidisciplinary team involved in this trial will ensure the successful completion of this and future trials.

##

## **Timeline**

| Submission of protocol to Pfizer Canada | Sept 2007 |
| --- | --- |
| Submission of protocol to Pfizer Global | Nov 2007 |
| Expected date of funding approval | Dec 2007 |
| Institutional REB approval, Contracts, Health Canada CTA | April 2008 |
| First patient entered | May 2008 |
| Final patient entered | July 2009 |
| Results available | Nov 2009 |

# 3. TRIAL OBJECTIVES

## **3.1. Primary Objective**

To define the safety, tolerability and maximum tolerated dose (MTD) of CP-675-206 in combination with radiation therapy. This trial is being conducted as a prelude to a planned Phase II trial of CP-675,206 in combination with radiation in metastatic breast cancer.

## **Secondary Objectives**

To monitor for evidence of clinical efficacy of CP-675-206 in combination with local radiation.

To monitor for evidence of immunological responses following administration of CP-675-206 in combination with local radiation.

# 4. TRIAL DESIGN

## **4.1. Overview**

This is a Phase 1, open-label, non-randomized dose escalation clinical trial evaluating the safety and tolerability of escalating doses of CP-675-206 combined with local radiation therapy in patients with incurable locally advanced or metastatic breast cancer. Subjects will receive one treatment cycle on this trial.

Subjects will be given 2000cGy of external radiation in 5 daily fractions at one tumor site using 6-18 MV photons. On the third day of radiation, subjects will also be given one dose of CP-675,206 intravenously.

Patients will be evaluated for toxicities at the following dose levels of CP-675,206: 3, 6, 10 and 15mg/kg. Dose escalation will proceed until the MTD is identified or up to the 15 mg/kg dose level. A minimum of six subjects will be treated with the MTD in order to characterize toxicities and responses.

The estimated maximum number of subjects that will be needed to achieve the trial objectives is 32 patients (range: 6-32 subjects; refer to section 10.2: Sample Size Determination).

## **Dose Escalation Rules**

To minimize the risk of this combination to patients, dosing in the first dose level cohort only will proceed by evaluating safety in the first patient for 12 weeks after the dose of CP-675,206. Prior CP-675,206 single-agent data on safety findings from Pfizer studies indicate that a 6-week follow-up interval was appropriate. However, in order to monitor for potential late-onset toxicities due to the combination of CP-675,206 with radiation, this protocol will follow patients for 12 weeks.

Dosing will be conducted in subsequent dose level cohorts of at least 3 patients, with patients assigned to a dose group after verification by the Investigator of eligibility.

Safety considerations in a current dose level will be assessed as follows to determine dose level escalation:

**1.** If no patient experiences a DLT in the first 12 weeks, the trial will proceed to the next dose level cohort

**2.** If 1/3 patients experiences a DLT in the first 12 weeks, expand cohort to 3 additional patients, and then determine:

- 1. • If 1/6 patients experiences a DLT in the first 12 weeks, escalate to the next dose level.
  2. • If ≥2/6 patients experience a DLT in the first 12 weeks, the current dose level will be considered above the maximum tolerated dose and dose escalation will be discontinued.

**3.** If ≥2/3 patients experience a DLT in the first 12 weeks, the current dose level will be considered above the MTD and the preceding dose level cohort will be expanded to a minimum of 6 patients.

The dose escalation will end after DLT is observed in ≥2 out of 3-6 patients treated at any dose level or after 6 patients at the highest dose level defined (CP-675,206, 15 mg/kg) have completed the 12-week observation period.

Additional patients may be entered as needed at any dose level, including intermediate dose levels, to more fully elucidate the pattern of toxicity and/or better define the MTD.

## **4.3. MTD Evaluable Patient - Definition**

All patients who are able to receive 1 dose of CP-675,206 and complete all 5 fractions of radiation will be deemed MTD evaluable.

Note: While patients will be considered MTD evaluable at the end of the 12-week observation period, dose escalation will occur only when all patients in the cohort have been observed for a full 12 weeks.

## **4.4. Dose Limiting Toxicities**

Adverse events and other symptoms will be graded according to the NCI Common Terminology Criteria for Adverse Events version 3.0. (<http://ctep.cancer.gov/reporting/ctc.html>).

Dose-limiting toxicity (DLT) is defined as any of the following when considered related to CP-675,206 therapy:

1. • Any CTC Grade 4 treatment related toxicity of any duration.
2. • Other Grade 3 or greater toxicities that do not recover to ≤ Grade 1 or baseline within 7 days of maximal management
3. • Grade 2 or greater treatment related clinical autoimmune toxicity of critical organs, which are defined as lung, heart, kidney, bowel, bone marrow and nervous system, including the eye (except anterior uveitis).

Assessment of DLTs during the first 12 weeks after CP-675,206 dosing will be completed to determine the next dose level.

## **Maximum Tolerated Dose**

The MTD will be the maximum dose level of CP-675,206 administered (3, 6, 10 or 15 mg/kg) in combination with radiation, at which no subject experiences a life-threatening adverse event and at which 0/6 or 1/6 patients (<33%) experiences a DLT during the first 12 weeks following CP-675,206 dose administration.

# 5. STUDY POPULATION

## **5.1. Inclusion criteria**

1. Inoperable locally recurrent or metastatic breast cancer not amenable to curative therapy.
2. ECOG PS 0-1.
3. Age >18 years.
4. Radiation therapy for palliation is medically indicated.
5. The patient does not have any contraindications to receiving the radiation therapy dose specified in this protocol.
6. There are no restrictions on previous lines of therapy. Prior chemotherapy is permitted but ≥4 weeks must have elapsed since the last dose.
7. Adequate organ function as defined by the following criteria:
8. Serum ALT ≤1.5 x upper limit of normal (ULN);
9. Total serum bilirubin ≤ 2xULN (patients with Gilbert’s Syndrome – direct serum bilirubin ≤2 x ULN);
10. Absolute neutrophil count (ANC) ≥ 1500/μL;
11. Platelets ≥100,000/μL;
12. Hemoglobin ≥11.0 g/dL for male or ≥ 10.0 g/dL for female;
13. Alkaline phosphatase ≤ 2 x ULN;
14. Serum LDH ≤ 2 x ULN;
15. Serum creatinine clearance ≥ 30ml/min;
16. Serum lipase/amylase ≤ 1.5 x ULN;
17. Serum magnesium within normal institutional limits;
18. TSH within normal institutional limits;
19. Patients must have an absolute baseline ejection fraction of ≥ institutional LLN by a gated MUGA or echocardiogram.
20. Able to sign and understand the informed consent document.

## **Exclusion criteria**

1. Previous treatment with any anti-CTLA4 agent.
2. Patients with active diarrhea at baseline.
3. Patients who will receive radiation to pelvic lesions (these patients will be excluded in order to minimize the risk of colitis).
4. History of chronic inflammatory or autoimmune disorder including, but not limited to the following: Addison’s disease, multiple sclerosis, Graves disease, Hashimoto’s thyroiditis, rheumatoid arthritis, systemic lupus erythromatosis, hypophysitis, pituitary disorders, pemphigus vulgaris, systemic mastocytosis, dermatomyositis/polymyositis, Sjörgen’s syndrome, vasculitis/arteritis, Behcet’s syndrome and psoriasis that is current or active in the last 3 years. Active vitiligo or a history of vitiligo will not be a basis for exclusion.
5. Patients with history of insulin-dependent diabetes.
6. History in the last 5 years of inflammatory bowel disease, celiac disease, or other chronic gastrointestinal conditions associated with diarrhea or bleeding not associated with the disease under study, or current acute colitis of any origin.
7. History (within the previous year) of congestive heart failure, stroke, myocardial infarction or thromboembolic event.
8. Patients with known brain metastases. Patients with clinical evidence suggestive of new brain metastases prior to enrollment are excluded if brain metastases have not been ruled out via CT or MRI. Patients with previously diagnosed brain metastases are eligible if they have completed their CNS treatment and have recovered from the acute effects of radiation therapy or surgery prior to the start of trial medication, have discontinued corticosteroid treatment for these metastases for at least 4 weeks, and are neurologically stable.
9. Patients of reproductive potential, who are not using an effective contraception, or who are pregnant or breastfeeding or have a positive (urine or serum) pregnancy test at baseline.
10. Concurrent or planned concurrent treatment with an immunosuppressive dose of corticosteroids or other immunosuppressive medication (eg, methotrexate, rapamycin) for longer than 10 days within 4 weeks prior to enrollment or while on-trial.
11. Any serious, uncontrolled medical disorder or uncontrolled active infection, including HIV or known or active viral hepatitis that would impair the ability to receive trial treatment and affect the risk/benefit ratio for the patient, in the judgment of the treating investigator.
12. Asymptomatic hepatitis C-positive patients would require a negative viral amplification by RT-PCR.
13. Psychiatric illness or social challenges that would preclude trial participation and compliance.

## **5.3. Enrolment criteria**

Following full assessment and determination that a patient meets all eligibility criteria and has given written informed consent for trial participation, the Investigator or designee will enroll the patient. Patients will be assigned individual patient identification numbers.

No patient shall receive investigational therapy until the entire registration process has been completed.

# TRIAL TREATMENTS

All patients being considered for the trial and eligible for screening must sign an informed consent form prior to any trial-specific procedures. Following completion of the pretreatment assessments and confirmation of eligibility, patients may be registered.

## **Trial Period**

Patients will receive radiation therapy and CP-675,206 on an outpatient basis. Radiation therapy will be given in 5 daily fractions. The radiation treatment can begin on either a Monday or a Tuesday. Clinic visits, hematology and other safety laboratory tests will be performed according to Table 2. Additional assessments may be performed as necessary to evaluate specific adverse events until they resolve to baseline or CTC Grade ≤1. Additional clinic visits may be required at the discretion of the investigator.

### **6.1.1. Dose Levels**

The starting dose of CP-675,206 will be 3.0 mg/kg, a dose shown to be safe as a single agent in previous trials. Dose escalation will only proceed to the 6, 10 and 15 mg/kg dose levels if adequate safety is demonstrated at the previous CP-675,206 dose level tested.

A minimum of 3 patients will be treated at each dose level prior to escalating to the next dose level. The decision to escalate to the next dose level will be made after the last additional patient on the current dose level has been observed through the first 12 weeks, in order to assess/rule out any dose limiting toxicity (DLT).

**CP-675,206 and radiation therapy: planned dose levels**

| Planned dose level | Radiation therapy | CP-675,206 dose  (given on the third day of radiation) |
| --- | --- | --- |
| 1  (starting dose) |  | 3 mg/kg |
| 2 | 2000cGy  (external, local radiation in 5 daily fractions) | 6 mg/kg |
| 3 |  | 10 mg/kg |
| 4 |  | 15 mg/kg |

### **6.1.2. Patient Dose Modifications (CP-675,206)**

#### 6.1.2.1. CP-675,206 dose adjustments due to changes in body weight

To adjust for possible fluctuations in body weight over time, patients will be weighed just prior to dosing, and the dose of CP-675,206 (re)calculated.

### **6.1.3. Patient Dose Modifications (Radiation Therapy)**

If the patient exhibits excessive toxicities, the last treatment or two may be omitted. The dose per treatment is not normally modified.

### **6.1.4. Management of CP-675,206 Toxicity**

CP-675,206 is an immune modulator, so Investigators should be alert for the development of immune-mediated side effects.

The majority of the adverse events reported in early phase trials have been manageable and well tolerated. The most common treatment-emergent, treatment-related adverse event is diarrhea. Diarrhea typically developed 1 to 2 months after the first dose of CP-675,206 (range: 4-72 days).

Although CP-675,206 is a fully human monoclonal antibody, immunogenicity remains a possibility, and thus acute hypersensitivity reactions such as urticaria, pruritus, laryngeal edema, angioedema, bronchospasm, respiratory distress, nausea, vomiting, abdominal cramps and pain, acute back pain, fever, diarrhea, hypotension and fatal anaphylactic shock, as well as sub acute reactions, are also possible. Possible immune-mediated disorders observed involve the skin (vitiligo and cutaneous leukocytoclastic vasculitis), the thyroid gland (autoimmune thyroiditis), the liver (autoimmune hepatitis), the pituitary (hypophysitis) and bone marrow (immune thrombocytopenic purpura). Abnormal lab results that may be immune-mediated include elevations of serum lipase and amylase and liver function tests. There has been one report of treatment-related uveitis, which resolved within 48 hours after treatment with topical steroids. Across all studies, toxicities generally recovered within 12 weeks, allowing continuation of treatment.

If a patient has an adverse event that is thought to be potentially immune-mediated (other than vitiligo), the Investigator should send a blood sample for autoimmune antibody testing.

CP-675,206 is a monoclonal antibody of the IgG2 type, and it is expected that it would cross the placenta if administered to a pregnant woman and could be secreted in breast milk of a lactating woman. The effect of exposure of a fetus or newborn to CP-675,206 is unknown. Women of childbearing potential must agree to use contraception during this trial and for 12 months after the last dose of CP-675,206.

*Note:* In general, it is not expected that the combination of CP-675,206 with this radiation dose will increase CP-675,206-related toxicities. However, radiation of the sites that are the most common autoimmune targets following CP-675,206 administration will be avoided when possible.

#### 6.1.4.1. Diarrhoea Management

Diarrhea is an expected clinically significant adverse event when treating patients with anti-CTLA4 investigational drugs. The diarrhea may be severe, may require hospitalization, and may result in intestinal perforation. The following algorithm is recommended for the treatment of patients who develop diarrhea while on trial:

| **Diarrhea Treatment Algorithm** | |
| --- | --- |
| **Diarrhea Severity** and Duration** | **Recommendation** |
| Grade 1 less than or equal to 14 days | • Consider use of probiotics  • Follow Diarrhea Management Guidelines including *C. difficile* titer, stool tests, empiric loperamide, oral fluid replacement  • Consider use of olsalazine, or masalamine |
| Grade 1 more than 14 days or Grade 2 of any duration not responsive to loperamide | • Consider use of probiotics  • Evaluation of severity by clinician familiar with CP-675,206-related diarrhea  • Consider IV fluids if indicated  • Consider use of budesonide, olsalazine, or masalmine  • Consider use of steroids* |
| Grade 3 or Grade 4 for any duration  Or  Any grade & duration associated with evidence of severe enterocolitis including bleeding, fever, pain or other signs/symptoms | • Consider use of probiotics  • Evaluation by clinician familiar with CP-675,206-related diarrhea  • Consider inpatient hospitalization for IV fluids, monitoring  • Consider use of budesonide, olsalazine, or masalamine  • Consider use of steroids* |
| ***** Response of diarrhea to steroids has been reported for a similar experimental drug that targets CTLA4. Example: oral or intravenous dexamethasone up to 4 mg every 4 hours  ** Grade 1 = increase of < 4 stools/d over baseline  Grade 2 = increase of 4-6 stools/d over baseline  Grade 3 = increase of ≥ 7 stools/d over baseline  Grade 4 = life-threatening consequences (e.g. hemodynamic collapse) | |

#### 6.1.4.2. Diarrhoea Prophylaxis

To date there is no data or evidence indicating effectiveness of prophylactic therapy for prevention of treatment emergent diarrhea secondary to CP-675,206. However, prophylaxis may be considered. If prophylaxis is used, one of the following drugs would be recommended:

1. • Probiotics (active cultures of beneficial bacteria which can be found in some brands of yogurt or in supplements) have been shown to be effective with other types of diarrhea, including ulcerative colitis, in clinical trials. Probiotics might prevent diarrhea by replacing gut bacteria with more benign strains, thereby decreasing the overactive T cell response to gut flora. This approach is not expected to present a risk to the patient.
2. • Mesalomine (Asocal) is an anti-inflammatory drug, and it takes several days to be effective. It may be considered for prophylactic use, but there is currently no data to support its use.

Loperamide treats only the symptoms of diarrhea and not the underlying cause. It should not be used prophylactically.

Steroids can be used to treat severe or prolonged diarrhea due to CP-675,206, but should not be used prophylactically.

### **6.1.5. Management of Hypersensitivity Reactions**

In case of hypersensitivity reactions, the Investigator should institute treatment measures deemed medically appropriate. The following treatment recommendations may be applicable and can be adopted at the Investigator’s judgment:

| **Hypersensitivity reaction:** | **Treatment recommendations:** |
| --- | --- |
| CTCAE v.3.0 Grade 1 Allergy (transient flushing or rash, drug fever <38°C) | • Supervise at the bedside. |
| CTCAE v.3.0 Grade 2 Allergy (urticaria, drug fever ≥ 38°C, and/or asymptomatic bronchospasm) | - 1. • Interrupt the infusion of CP-675,206 and disconnect infusion tubing from patient;   2. • Administer IV antihistamines (diphenhydramine, 25-50 mg, and ranitidine, 50 mg, or cimetidine, 300 mg);   3. • After recovery of symptoms, resume the infusion at half the initial infusion rate. If no further symptoms appear, complete the administration of the dose. If symptoms reappear, stop infusion and discontinue patient from the trial. |
| CTCAE v.3.0 Grade 3 or 4 Allergy (symptomatic bronchospasm requiring parenteral medication(s) with or without urticaria; allergy-related edema/angioedema; hypotension; anaphylaxis) | - 1. • Stop the infusion of CP-675,206 and disconnect infusion tubing from patient;   2. • Administer epinephrine (1:10,000) in 3.5 to 5 mL IV boluses (no more than 6 doses);   3. • Administer IV antihistamine (diphenhydramine 50 mg IV push);   4. • If wheezing persists: 0.35 mL of nebulized albuterol or other bronchodilators;   • Consider methylprednisolone 30 to 60 mg IV push, which may prevent recurrent or ongoing reactions; |

## **6.2. CP-675,206 Drug supply**

### **6.2.1. CP-675,206 - Preparation and Dispensing**

It is recommended that appropriate site pharmacy personnel (or equivalent) refer to the most current version of the Dosing and Administration Instructions (DAI) for detailed instructions on the preparation and administration of CP-675,206. It is suggested that all manipulations are carried out in a laminar flow cabinet using aseptic technique for maximum patient protection. Only the necessary materials should be present in the working area during each preparation step.

### **6.2.2. Administration**

The investigational drug will be administered as specified in the most current version of the DAI. CP-675,206 administration will take place in the Princess Margaret Hospital chemo daycare unit which has extensive experience with drug administration and management of toxicities.

Although CP-675,206 is a fully human monoclonal antibody, immunogenicity remains a possibility, and thus acute hypersensitivity reactions such as urticaria, pruritus, laryngeal edema, angioedema, bronchospasm, respiratory distress, nausea, vomiting, crampy abdominal pain, acute back pain, fever, diarrhea, hypotension and fatal anaphylactic shock, as well as subacute reactions, are also possible. The patients’ blood pressure, heart rate, and temperature should be recorded prior to treatment and monitored, as needed during drug infusion and for approximately 1 hour post- infusion.

Medications to treat hypersensitivity reactions should be available, such as IV saline, acetaminophen, and emergency drugs, including subcutaneous epinephrine, diphenhydramine, methylprednisolone, and nebulized albuterol.

## **Concurrent Medication(s)**

Once a patient begins trial treatment, the addition of any other concurrent cancer treatment (including cytotoxic, hormonal, homeopathic, or immunologic therapy) might confound the assessment of safety and efficacy and, therefore, is not recommended. Such concurrent treatments should be delayed for 12 weeks following CP-675,206 dosing if clinically acceptable. Due to the unknown potential for drug-drug interactions, the use of any unapproved, naturalistic, homeopathic, or holistic therapies is disallowed. Medication intended solely for supportive care (eg, analgesics, antibiotics, antiemetics, antidiarrheals, or antidepressants) may be used at the investigator’s discretion. During the trial, patients may require immunosuppressive drugs such as corticosteroids for management of underlying disease, treatment-related toxicity, or unrelated conditions.

Steroid use for no longer than 10 days is permitted. However low dose daily steroid use (5 mg of prednisone or equivalent daily) and topical and inhaled corticosteroids in standard doses are allowed.

It is highly recommended that patients not be exposed to anti-infective vaccinations while participating in the trial (i.e. within the minimum observation period of 12 weeks following CP-675,206 dosing), given that the effect of the vaccination has not been explored under conditions of CTLA4 blockade. Any such vaccinations will be recorded on the Concomitant Medication CRF.

## **Salvage Therapy**

Patients who discontinue treatment on this trial may receive subsequent therapy based on the judgment of the treating physician.

## **Additional cycles**

Patients that exhibit a clinical response following CP-675,206 dosing may be eligible to receive a subsequent dose of CP-675,206 90 days following the first dose. Additional dosing will be based on the judgment of the treating physician and after communication with Pfizer.

# 7. TRIAL PROCEDURES

The minimum required screening, on-study and follow-up evaluations are summarized in the following sections. Other parameters and/or increased frequency of examinations or clinical follow-up may be needed based on the findings during the trial.

Informed consent must be obtained from each patient being considered for the trial before any protocol-specified procedure is performed. In the event of scheduling conflicts, trial evaluations will take place on the designated day ± 3 days.

## **Pre-trial Assessments**

The minimum required screening procedures must be performed within 14 days before enrollment (unless otherwise specified). Screening procedures must be fully documented in the patient’s medical record and transferred to the CRF. Pretrial Assessments are outlined below:

**Table 1. Pre-trial assessments**

| **Assessment/Test** | **Within 72 Hours** | **Within 5 days** | **Within 14 Days** | **Within 28 Days** |
| --- | --- | --- | --- | --- |
| Obtain Informed Consent |  |  | X |  |
| Medical History^1^ |  |  | X |  |
| Baseline Signs & Symptoms^2^ |  |  | X |  |
| Enrollment |  | X |  |  |
| Height |  |  | X |  |
| Weight |  |  | X |  |
| Physical Exam, including vital signs and clinical signs of autoimmunity^3^ |  |  | X |  |
| Review of Concomitant Medications^4^ |  |  | X |  |
| ECOG status |  |  | X |  |
| ECG |  |  | X |  |
| Pregnancy Test (serum or urine) for women of childbearing potential (WOCBP)^5^ | X |  |  |  |
| Hematology^6^ |  |  | X |  |
| Serum Chemistry^7^ |  |  | X |  |
| Total IgG |  |  | X |  |
| Microscopic Urinalysis^8^ |  |  | X |  |
| Thyroid Function^9^ |  |  | X |  |
| HIV, Hepatitis screening^10^ |  |  | X |  |
| HLA Typing |  |  | X |  |
| Adverse Events |  |  | X |  |
| Tumor Imaging (CT, MRI)^11^ |  |  |  | X |

**1.** Includes review of systems, oncologic history and general medical history of all disease processes (active or resolved), and concomitant illnesses. Disease characteristics include diagnosis, date of diagnosis, cytology or histology, stage at time of trial entry, and breast cancer treatment history.

**2.** The Investigator should describe any signs and symptoms at presentation reported by patients during the 14 days prior to the initial dose of CP-675,206. Patients should be questioned about visual symptoms. Any patient who is experiencing symptoms suggestive of uveitis or melanoma-associated retinopathy (eg eye pain or redness, sensitivity to light or glare, blurred vision, floaters, night blindness, or visual field defects) should be evaluated by an ophthalmologist to rule out these conditions.

**3.** The physical exam should be performed by an Investigator at baseline, and a physician or designee may perform subsequent assessments, as appropriate. Vital signs include temperature, blood pressure, heart rate while sitting

**5.** Must be negative within 72 hours prior to the initial dose of CP-675,206.

**4.** All concomitant medications taken within 14 days need to be documented and recorded (including start and stop dates, as appropriate) on the patient CRF.

**6.** Hemoglobin, WBC with 5-part differential (percent and absolute values), platelet count.

**7.** Calcium, chloride, total protein, potassium, random glucose, sodium, blood urea nitrogen/BUN, creatinine, AST/SGOT, ALT/SGPT, alkaline phosphatase/ALP, lactic acid dehydrogenase/LDH, amylase, lipase, albumin, total bilirubin

**8.** Collect urine for Urinalysis [must include: 1) microscopic analysis of urinary sediment and 2) analysis for protein, glucose, and blood (other, as indicated)].

**9.** Thyroid stimulating hormone (TSH), T3, T4.

**10.** Refer to Exclusion Criteria.

**11.** Documentation of Baseline target and non target lesions by imaging techniques or by measurement of clinical lesion(s) must be performed within 28 days prior to dosing. The method of tumor assessment (CT, MRI) and tumor sites are to be consistent from baseline to the end of the study.

## **7.2. On-Trial Assessments**

One to two days prior to CP-675,206 dosing (i.e. day 1 or 2 of radiation treatment), the following should be done and results available prior to day 3 CP-675,206 administration:

1. **1.** All women of childbearing potential must have a negative serum or urine pregnancy test within 72 hours prior to the initial dose of CP-675,206 (see “Pre-trial assessments”).
2. **2.** Collect urine for Urinalysis [must include: 1) microscopic analysis of urinary sediment and 2) analysis for protein, glucose, and blood (other, as indicated)].
3. **3.** Blood draws for hematology, serum chemistry, autoantibodies.
4. **4.** Other blood draws will also be done but results do not need to be available prior to CP-675,206 administration (HAHA, immune monitoring).

On the day of CP-675,206 dosing, the following should be done prior to the dose of CP-675,206:

1. **1.** Baseline Signs and Symptoms
2. **2.** Review of Concomitant Medications
3. **3.** ECOG Performance Status/Weight
4. **4.** Clinical Assessments/Vital Signs (temperature, blood pressure while sitting, heart rate)
5. **5.** Physical Exam
6. **6.** Review of visual symptoms
7. After the assessments listed above are performed and safety labs reviewed, investigational drug may be administered intravenously. During infusion, vital signs will be monitored and treatment-emergent adverse events will be recorded.

All other tests and assessments to be performed during treatment with CP-675,206 are outlined in the following table.

**Table 2. On-trial assessments**

**Test/Assessment: Study Day Number:**

|  | D1 | D2 | D3 | D4 | D5 | D8  (1w) | D15  (2w) | D22  (3w) | D29  (4w) | D43  (6w) | D57 (8w) | D71  (10w) | D85  (12w) |
| --- | --- | --- | --- | --- | --- | --- | --- | --- | --- | --- | --- | --- | --- |
| Weight |  |  | X |  |  | X | X | X | X | X | X | X | X |
| Physical Exam, including signs of autoimmunity |  |  | X^1^ |  |  | X | X | X | X | X | X | X | X |
| ECOG status |  |  | X^1^ |  |  | X | X | X | X | X | X | X | X |
| Hematology^2,3,4^ | X |  |  |  |  | X | X | X | X | X | X | X | X |
| Serum Chemistry^3,4,5^ | X |  |  |  |  | X | X | X | X | X | X | X | X |
| Microscopic or Dipstick Urinalysis^3,4,6^ | X |  |  |  |  | X | X | X | X | X | X | X | X |
| Thyroid Function^3,4,7^ |  |  |  |  |  |  |  |  | X |  |  |  | X |
| Autoantibody Panel^3,4,8^ | X |  |  |  |  |  |  |  | X |  |  |  | X |
| Human anti-human antibody (HAHA)^9^ | X |  |  |  |  |  |  |  | X |  |  |  | X |
| Radiation^10^ | X | X | X | X | X |  |  |  |  |  |  |  |  |
| CP-675,206 Administration |  |  | X |  |  |  |  |  |  |  |  |  |  |
| Immune Monitoring^11^ | X |  |  |  |  | X | X |  | X |  | X |  | X |
| Blood specimen for genomic analysis^12^ | X |  |  |  |  |  |  |  |  |  |  |  |  |
| AE Monitoring^13^ | X | X | X | X | X | X | X | X | X | X | X | X | X |
| Tumor Imaging (CT, MRI)^14^ |  |  |  |  |  |  |  |  |  |  | X |  |  |

**1.** Tests performed/specimens drawn prior to dosing with CP-675,206.

**2.** Hemoglobin, WBC with 5-part differential (percent and absolute values), platelet count

**3.** Clinically significant abnormalities should be reported as Adverse Events in the CRF.

**4.** Assessments may be performed within 24 hours of the designated timepoints.

**5.** Calcium, chloride, total protein, potassium, random glucose, sodium, blood urea nitrogen/BUN, creatinine, AST/SGOT, ALT/SGPT, alkaline phosphatase/ALP, lactic acid dehydrogenase/LDH, amylase, lipase, albumin, total bilirubin

**6.** Complete urinalysis (with microscopic analysis of centrifuged sediment for casts or other abnormalities); performed at baseline. In subsequent evaluations, dipstick urine testing is allowed in lieu of microscopic analysis, however, microscopic analysis must be performed if dipstick results are abnormal.

**7.** Thyroid stimulating hormone (TSH), T3, T4. Thyroid function tests are also done as part of the pre-trial assessments (within 14 days before treatment begins).

**8.** The autoantibody panel will include antinuclear antibodies (ANA), antineutrophil cytoplasmic antibodies (ANCA), anti-DNA antibody (SS and DS), Rh factor (RhF), antithyroglobulin, anti liver-kidney microsomal (LKM), anti-islet-cell, antibodies to Ro (SSA) and to La (SSB) and antiphospholipid and/or other autoantibody tests.

**9.** Human anti-human antibody may be done at the Investigator’s discretion.

**10.** A total of 2000cGy to be delivered in 5 daily fractions using 6-18 MV photons.

**11.** Immune monitoring will be done using peripheral blood obtained by phlebotomy. PBMC and serum will be isolated and cryopreserved.

**12.** DNA extraction and banking for genomic evaluation.

**13.** Adverse event assessment to be performed continuously throughout the trial. Treatment-related adverse events will be followed until they resolve to baseline or Grade 1 or until the treating physician determines that they are irreversible.

**14.** Patients with measurable disease will be assessed by imaging every 8 weeks until progression (range 6-8 weeks). If a response is seen at week 8, the imaging will be repeated 4 weeks later. The same method of assessment and the same technique should be used for repeated measurements.

## **7.3. Follow-up Visits**

### **7.3.1. Treatment-Related Adverse Event Follow-Up**

Approximately one month (30 days) after the end of treatment evaluation, a follow up visit is required. Such on going AEs should be followed to ensure that they have resolved, returned to baseline, or are deemed irreversible by the Investigator. If there is evidence of continuing CP-675,206-related toxicity, the patient should continue to be followed at intervals deemed medically appropriate by the Investigator. The required (minimum) laboratory tests and observations for these patients are the following:

- 1. Physical exam, including signs of autoimmunity.
  2. If clinically warranted: hematology, serum chemistry, urinalysis, autoantibody panel.
  3. Treatment-related adverse events will be followed to resolution or until the treating physician determines that they are irreversible.

Additional observations and laboratory tests should be performed according to medical need.

### **7.3.2. Disease Progression Follow-Up**

All patients will be followed for disease progression (if prior SD, PR, or CR) or until the start of a new therapeutic regimen for breast cancer, whichever comes first. This information will be recorded in the CRF. It is understood that not all patients will be available for continued clinic visits; however, every attempt should be made to collect progression date and subsequent therapy (start of therapy).

### **7.3.3. Survival Follow-Up**

All patients should continue to be followed every 3 months when possible for up to 1 year. It is understood that not all patients will be available for continued clinic visits; however, every attempt should be made to access survival information (disease status, alive or dead, and date of death). This information may be obtained by telephone interview if the patient is unable to visit the clinic.

## **Patient Withdrawal**

Patients may withdraw from the trial at any time at their own request, or they may be withdrawn at any time at the discretion of the Investigator for safety, behavioral, or administrative reasons. If a patient does not return for a scheduled visit, every effort should be made to contact the patient. In any circumstance, every effort should be made to document patient outcome, if possible. The Investigator should inquire about the reason for withdrawal, request the patient to return all unused investigational product(s), request the patient to return for a final visit, if applicable, and follow-up with the patient regarding any unresolved adverse events.

If the patient withdraws from the trial and also withdraws consent for disclosure of future information, no further evaluations should be performed and no additional data should be collected. The Investigator may retain and continue to use any data collected before such withdrawal of consent.

A discontinuation occurs when an enrolled patient ceases participation in the trial, regardless of the circumstances, prior to completion of the protocol. The Investigator must determine the primary reason for discontinuation:

1. **1.** Withdrawal due to adverse event. When a discontinuation is due to a serious adverse event (SAE), the serious adverse event must be reported in accordance with the reporting requirements.
2. **2.** Patients may decide to withdraw from the trial at any time. Patients who withdraw from treatment should be followed for survival, and their subsequent treatments should be recorded.
3. **3.** Patients must be discontinued if the Investigator believes it to be in the patient’s best interest to begin chemotherapy or biological therapy for his/her disease.
4. **4.** The Investigator should withdraw the patient at any time if he/she believes it is in the patient’s best interest to do so.

The final evaluation required by the protocol will be performed at the time of trial discontinuation. The Investigator will record the reason for trial discontinuation and provide or arrange for appropriate follow-up (if required) for the patient.

# 8. STUDY ASSESSMENTS

## **8.1. Safety Assessments**

The safety of this protocol will be assessed by complete physical examination, assessment of hematology and coagulation function, serum chemistry, urinalysis, thyroid function and autoantibody panel.

## **8.2 Clinical response assessments**

All patients who have qualifying measurable disease outside the radiation field according to RECIST criteria, received investigational treatment, had baseline assessments and at least one on-study tumor assessment will be considered evaluable for response. Data on the response of the irradiated lesion will also be collected.

Tumor assessments will be performed at screening/baseline and then at 6-8 weeks following CP-675,206 dosing. Subjects exhibiting stable disease, partial response or complete response at the 8 week timepoint will undergo confirmatory re-evaluation 4-6 weeks following the first documented response. Patients may also be evaluated at 12 weeks following treatment. The same method of assessment and the same techniques used at screening must be performed.

Patients who were treated and removed from the trial prior to on-study tumor assessment for reasons such as patient request, lack of compliance, or as early toxicity (patients who went off study due to serious adverse events related to the trial drug therapy prior to reassessment of tumor lesions) will not be considered evaluable for the secondary objective of efficacy of the trial. Patients who discontinue treatment with stable disease or an unconfirmed response, who subsequently have a response confirmed prior to starting new treatment, will be classified as responders. If no confirmation is obtained, they will be considered as non-responders.

### **8.2.1. Measurability of Tumor Lesions**

Measurements of all lesions should be recorded in metric units. All baseline evaluations must be performed as close as possible to the first day of trial treatment and, in the case of patients with evidence of disease, never more than 4 weeks (28 days) before enrollment. The same method of assessment and the same technique should be used to characterize each identified and reported lesion at baseline and during follow-up.

Measurable Lesions

• If the measurable disease is restricted to a solitary lesion, its neoplastic nature could be confirmed by cytology/histology if necessary.

• Lesions that can be accurately measured in at least 1 dimension (longest diameter to be recorded) as ≥2.0 cm with conventional techniques or ≥1.0 cm with spiral CT scan.

• Clinical lesions will only be considered measurable when they are superficial (eg, skin nodules). Clinically detected lesions will only be considered measurable when they are superficial (eg, skin nodules) and the longest diameter is ≥2 cm. Palpable lymph nodes ≥2.0 cm should be demonstrable by CT scan.

Non-measurable Lesions

• All other lesions, including small lesions (longest diameter <2.0 cm with conventional techniques or <1.0 cm with spiral CT scan) and truly nonmeasurable lesions. Also, skin lesions with longest diameter <1.0 cm and other clinical lesions <2.0 cm. Lesions that are considered nonmeasurable include bone lesions, leptomeningeal disease, ascites, pleural/pericardial effusion, inflammatory breast disease, lymphangitis cutis/pulmonis, and abdominal masses that are not confirmed and followed by imaging techniques. Previously irradiated lesions are nonmeasurable except in cases of documented progression of the lesion since the completion of radiation therapy.

### **8.2.2. “Target” and “Non-Target” Lesions**

Baseline tumor measurements must be performed not more than 4 weeks (28 days) prior to the initiation of treatment with CP-675,206. Patients must have at least one measurable lesion outside of the radiation field at baseline to be included in the analysis of this endpoint. If the measurable disease is restricted to a solitary lesion, its neoplastic nature could be confirmed by cytology/histology if necessary.

Baseline documentation of tumor sites may include imaging assessment of disease in the chest, abdomen and pelvis. A baseline imaging study of the brain is required.

Target Lesions

• Up to 10 total lesions, a maximum of 5 lesions per organ, that are representative of all involved organs may be selected and recorded as target lesions at baseline. Target lesions should be selected on the basis of their size (lesions with the longest diameter) and their suitability for accurate repetitive measurements (either by imaging techniques or clinically). A non-measurable lesion cannot be selected as a target. A sum of the longest diameter (LD) for *all target lesions* will be calculated and reported as the baseline sum LD. The baseline sum LD will be used as reference to further characterize the objective tumor response of the disease.

Non-Target Lesions

• All other lesions (or sites of disease) should be identified as non-target lesions and recorded as non-target lesions at baseline. Measurement of non-target lesions at baseline is not required and should be recorded as “present.” Each non-target lesion should be documented as either present, absent or new in each subsequent evaluation.

### **8.2.3. Techniques for Assessing Measurable Disease**

The same method and technique of assessment used at baseline should be used to characterize each identified and reported lesion during follow-up. Imaging-based evaluation is preferred to evaluation by clinical (physical) examination when both methods have been used to assess the antitumor effect of a treatment.

• Accepted methods of tumor assessment include: Clinical Examination: Clinical lesions will only be considered measurable when they are superficial (eg, skin nodules). Clinically detected lesions will only be considered measurable when they are superficial (eg, skin nodules) and the longest diameter is ≥2 cm. Palpable lymph nodes ≥2.0 cm should be demonstrable by CT scan.

• Chest X-ray (CXR): Lesions on CXR are acceptable as measurable lesions when they are clearly defined and surrounded by aerated lung. However, computerized tomography (CT) is preferable.

• CT, magnetic resonance imaging (MRI): Conventional CT and MRI should be performed with contiguous cuts of 10 mm or less in slice thickness. Spiral CT should be performed using a 5 mm contiguous reconstruction algorithm. This applies to tumors of the chest, abdomen, and pelvis. Head and neck and extremities require specific protocols as per RECIST.

**• Ultrasound:** Ultrasound should not be used to measure tumor lesions not clinically accessible when the primary endpoint is objective response. It is, however, a possible alternative to clinical measurements of superficial palpable nodes, subcutaneous lesions, and thyroid nodules. Ultrasound may be used to confirm the complete disappearance of superficial lesions usually assessed by clinical examination.

### **8.2.4. Objective Response Classifications**

The following RECIST criteria will be the primary method utilized in this trial for the assessment and reporting of tumor response data:

- Complete Response (CR): Disappearance of all target and non-target lesions. CR must be confirmed by repeat assessments performed no less than 4 weeks after the criteria for response are first met to qualify as CR;
- Partial Response (PR): At least a 30% decrease in the sum of the longest diameter (LD) of target lesions taking as reference the baseline sum LD. Non-target lesions may persist provided there is no unequivocal progression in these lesions.
- PR must be confirmed by repeat assessments performed no less than 4 weeks after the criteria for response are first met to qualify as PR;
- Progressive Disease (PD): At least a 20% increase in the sum LD of the target lesions from the smallest sum LD recorded since the beginning of therapy or the appearance of one or more new lesions or unequivocal progression of existing non-target lesions; and
- Stable Disease (SD): Measurements demonstrating neither sufficient shrinkage to qualify for PR nor sufficient increase to qualify as PD after the start of treatment taking as reference the smallest sum LD since the treatment started. During this time, non-target lesions may persist provided there is no unequivocal progression in these lesions.

### **8.2.5. Frequency of Tumor Measurements**

Tumor assessments will be performed every 6 to 8 weeks. CR and PR must be confirmed by repeat assessments performed 4-6 weeks after the criteria for response are first met to qualify as such response. Additional tumor measurements may be completed as needed.

### **8.2.6. Evaluation of Overall Response**

The best overall response is the best response recorded since the start of treatment until disease progression or recurrence (taking as reference for PD the smallest measurement since treatment started). For CR and PR the best response assignment will depend on the achievement of both the initial establishment and confirmation of CR or PR.

To be assigned a status of Partial Response (PR) or Complete Response (CR), changes in tumor measurements must be confirmed by repeat assessments that should be performed no less than 4 weeks after the criteria for response are first met, per RECIST. If a patient does not achieve a PR or CR but maintains Stable Disease (SD) for at least 10 weeks or more, then the best overall response for the patient will be SD.

| Overall Response | | | |
| --- | --- | --- | --- |
| Target Lesions | Nontarget lesions | New Lesions | Overall response |
| CR a | CR | No | CR |
| CR | Non-CR/Non-PD | No | PR |
| PR b | Non-PD | No | PR |
| SD c | Non-PD | No | SD |
| PD d | Any | Yes or No | PD |
| Any | PD | Yes or No | PD |
| Any | Any | Yes | PD |
| a, Complete Response  b, Partial Response  c, Stable Disease  d, Progressive Disease | | | |

##

## **8.3 Immunological monitoring**

Immunological monitoring will be done on peripheral blood mononuclear cells (PBMC) and serum using methods such as flow cytometric analysis and ELISA, respectively. Immunological responses will be evaluated at baseline and at 1, 2, 4, 8 and 12 weeks following CP-675,206 dosing. Additional time points may also be evaluated at the Investigator’s discretion. Samples will be cryopreserved until analysis.

The relative proportion of various T cell subsets in PBMC obtained by phlebotomy will be monitored by flow cytometry, as well as the expression of lymphocyte activation markers. For HLA-A*0201+ subjects, further assessments may be done in order to evaluate the frequency of T cells specific for defined breast tumor antigens. This may be done using MHC-peptide multimers and/or ELISpot assays. PBMC obtained from similar patients receiving irradiation alone will also be assayed to provide information on the immunological effect of irradiation alone. Serum will also be collected for monitoring responses to the investigational treatment, such as alterations in circulating cytokine levels or markers for epithelial cell apoptosis/necrosis by ELISA.

# 9. ADVERSE EVENT REPORTING

This study will utilize the CTCAE 3.0 for toxicity and Adverse Event reporting. A copy of the CTCAE 3.0 can be downloaded from the CTEP home page (http://ctep.cancer.gov). All appropriate treatment areas should have access to a copy of the CTCAE 3.0.

This study will be monitored by the Princess Margaret Hospital Data Safety Monitoring Board.

## **Reporting requirements**

Within one (1) working day, report by **telephone** to the Principal Investigator or research nurse serious adverse events as defined below. These events should also be reported within three (3) working days, in **writing**, to the Principal Investigator. An appropriate adverse event form should be used.

*Associated with the use of the drug/biologic*: There is a reasonable possibility that the experience may have been caused by the drug/biologic.

*Life threatening adverse drug/biologic experience*: Any adverse drug/biologic experience that places the subject, in the view of the investigator, at immediate risk of death from the reaction as it occurred.

*Serious adverse drug/biologic experience*: Any adverse drug/biologic experience occurring at any dose that results in any of the following outcomes:

- Death (including all deaths within 30 days of the last administration of study drug/biologic.

- A life-threatening adverse drug/biologic experience

- Inpatient hospitalization or prolongation of existing hospitalization

- A persistent or significant disability/incapacity

- A congenital anomaly/birth defect

- Any other medical event that, in appropriate medical judgment, may require medical or surgical intervention to prevent one of the outcomes listed above.

*Unexpected adverse drug/biologic experience*: Any adverse drug/biologic experience, the specificity or severity of which is not consistent with the product monograph, or not consistent with the risk information described above as a protocol-specific expected adverse event (see “Expected Adverse Events and Protocol-Specific Expedited Adverse Event Reporting Exclusions”, above).

Qualifying serious adverse events must be reported to the UHN Research Ethics Board (REB) according to the UHN REB’s required timeframe.

The Sponsor (IND/CTA holder) will be responsible for reporting all adverse events to the appropriate regulatory agencies (e.g. REB/Health Canada as applicable) as per each agencies individual reporting requirements and within each agencies required timeframe.

Data on all adverse experiences/toxicities (excluding fever and hematologic toxicities), regardless of seriousness must be collected for documentation purposes only. AEs should be followed for 6 weeks after the last dosing of study drug/biologic.

Non-serious adverse events will be reported to the appropriate regulatory agencies (e.g. REB/Health Canada as applicable) at the time of the annual report if required by that agency.

# 10. DATA ANALYSIS/STATISTICAL CONSIDERATIONS

## **10.1. Trial Populations**

The following population will be assessed: An as-treated population, defined as all patients enrolled in the trial that received radiation therapy and one dose of CP-675,206.

## **10.2. Sample Size Determination**

A minimum of 3 patients will be treated at each dose level prior to escalating to the next dose level, and a minimum of 6 patients will be studied at the MTD to determine its suitability as the recommended dose for Phase 2 combination studies. For details, please refer to Section 4.2, “Dose Escalation Rules”. An estimated maximum of 6 patients will be enrolled at each of 4 dose levels, plus an additional maximum of 6 patients for further evaluation at the MTD (for a total of 30 subjects). Additional patients may be entered as needed at any dose level, including intermediate dose levels. Overall, the sample size is determined empirically and it is expected that approximately 6-32 patients will be enrolled in this trial (allowing for 2 non-evaluable subjects).

## **10.3. Dose Escalation**

The operating characteristics of the dose escalation portion of this trial design are shown in the table below, which provides the probability of escalation to the next higher dose for each underlying true DLT rate. For example, for a DLT that occurs in 10% of patients, there is a greater than 90% probability of escalating. Conversely, for a DLT that occurs with a rate of 70%, the probability of escalating is 3%. (It is assumed that dose escalation occurs with either 0/3 or 1/6 patients with DLT).

| **Probability of Escalating Dose** | | | | | | | | | |  |
| --- | --- | --- | --- | --- | --- | --- | --- | --- | --- | --- |
| True Underlying DLT Rate | 10% | 20% | 30% | 40% | 50% | 60% | 70% | 80% | 90% | |
| Probability of Escalating Dose | 0.91 | 0.71 | 0.49 | 0.31 | 0.17 | 0.08 | 0.03 | 0.009 | 0.001 | |

The probability of failing to observe toxicity (such as DLT) in a sample size of 3 or 6 patients given various true underlying toxicity rates is shown below. For example, with 6 patients, the probability of failing to observe toxicity occurring at least 40% of the time is less than 5%.

| **Probability of Failing to Observe Toxicity** | | | | | | | | | |  |
| --- | --- | --- | --- | --- | --- | --- | --- | --- | --- | --- |
| True Underlying Toxicity Rate | 10% | 20% | 30% | 40% | 50% | 60% | 70% | 80% | 90% | |
| Probability Failing to Observe Toxicity, N=3 | 0.73 | 0.51 | 0.34 | 0.22 | 0.13 | 0.064 | 0.027 | 0.008 | 0.001 | |
| Probability Failing to Observe Toxicity, N=6 | 0.53 | 0.26 | 0.12 | 0.047 | 0.016 | 0.004 | <0.001 | <0.001 | <0.001 | |

##

## **10.4. Analysis of Primary Endpoint**

### **10.4.1. Safety Analysis**

Overall safety profile and toleration of CP-675,206 and irradiation will be characterized by type, frequency, severity (as graded by version 3.0 of the NCI CTCAE), timing and relationship of adverse events to investigational therapy and laboratory abnormalities.

## **10.5. Analysis of Secondary Endpoints**

### **10.5.1. Efficacy Analyses**

#### 10.5.1.1. Best Overall Response

For patients with measurable disease, best overall response will be defined according to the Response Evaluation Criteria in Solid Tumor (RECIST) guidelines. For patients without measurable disease, best overall response can be a CR (no evidence of disease), PR (eg unequivocal progression in non-measurable lesions or new lesion), or SD in all other cases. Listing of patients with their measurability status at baseline and best overall response on study will be provided.

#### 10.5.1.2. Duration of Tumor Response

Duration of Objective Response (CR or PR) will be measured from the date that a CR or PR is first documented (whichever occurs first) to date of progression or death due to progressive disease, whichever occurs first.

Duration of Complete Response will be measured from the date that a CR is first documented to date of progression or death due to progressive disease, whichever occurs first.

Patients last known to be progression-free are censored at the date they were last known to be progression-free, with the following exceptions.

**1.** Patients who go off treatment prior to progression will continue to be followed for progression until a new treatment is initiated. The date of new treatment will be used as the progression date unless the patient has clearly not progressed by the time of new treatment, as judged by the Investigator. If the patient has clearly not progressed, censorship will be at the date of the new treatment.

**2.** Patients who die of causes clearly not related to disease before progression, as judged by the Investigator, will be censored at date last known to be progression-free. Otherwise date of death will be used as the progression date.

#### 10.5.1.3. Progression-free Survival

Progression-free survival (PFS) is defined as the time from the date of enrollment to the date of first documentation of disease progression, or to the death from any cause. PFS data will be censored on the day following the date of last tumor assessment documenting absence of progressive disease for patients who do not have objective tumor progression and are still on trial at the time of an analysis, are given antitumor treatment other than the trial treatment, or removed from trial follow-up prior to documentation of objective tumor progression. Kaplan-Meier method will be used to obtain median PFS.

### **10.5.2. Analysis of Other Endpoints**

#### 10.5.2.1. Immune monitoring

Summary statistics (mean, median, standard deviation, quartiles, minimum and maximum, etc.) will be provided for evaluations of immunological responses as appropriate. These data will also be used to explore any correlation with the dose of CP-675,206 and clinical responses.

### **10.5.3. Interim Analysis**

An interim analysis of safety is planned after the second dose level is complete.

# 11. DATA HANDLING AND RECORD KEEPING

All source data and documents generated in their trial will be made available to trial-related monitoring, audits, Research Ethics Boards (REB) review and regulatory inspections as required.

## **Retention of Patient Records and Study Files**

The Therapeutic Products Directorate of Health Canada states in its *Good Clinical Practice: Consolidated Guidelines (ICH Guidance Document E6)* (1997) that the investigator and sponsor shall retain study records relating to the study until at least 2 years after the last approval of a marketing application and until there are no pending or contemplated marketing applications, or at least 2 years have elapsed since the formal discontinuation of clinical development of the investigational product. In the event of a trial discontinuation, sponsor records should also be kept for a minimum of 2 years. Records contained in the Clinical Trial Application should be maintained on file for at least 25 years. We will comply with these regulations.

#

# 12. ETHICS

## **12.1. Research Ethics Board Approval for Protocols**

*Initial Approval.* The protocol and consent forms will receive full ethics board approval by the University Health Network REB prior to commencement of the clinical trial.

*Continuing Approval.* Annual re-approval will be sought for as long as the trial is open to patient accrual.

*Amendments.*  An amendment to a protocol which constitutes a significant change will be submitted to review/approval by local REBs and, if applicable, by the Biological and Genetic Therapies Directorate of Health Canada, as required.

## **Informed Consent**

*Informed consent document.* The REB of an institution must approve the consent form that will be used at that centre prior to its local activation; changes to the consent form in the course of the study will also require REB notification/approval.

The following elements must appear in the consent form: a description of the purpose of the study (indicating, if appropriate, that the therapy is investigational); potential side effects, potential benefits; study design; voluntary participation; and confidentiality. It is essential that the consent form contain a clear statement which gives permission for 1) information to be sent to and 2) source medical records to be reviewed by government agencies as necessary. The consent forms attached to this document meet these requirements.

*Consent process/patient eligibility*. Patients who cannot give informed consent (i.e. mentally incompetent patients, or those physically incapacitated such as comatose patients) are not to be recruited into the study. Patients competent but physically unable to sign the consent form may have the document signed by their nearest relative or legal guardian. Each patient will be provided with a full explanation of the study before consent is requested.

# 13. TABLES

## **Table 1. Pre-trial assessments**

| **Assessment/Test** | **Within 72 Hours** | **Within 5 days** | **Within 14 Days** | **Within 28 Days** |
| --- | --- | --- | --- | --- |
| Obtain Informed Consent |  |  | X |  |
| Medical History^1^ |  |  | X |  |
| Baseline Signs & Symptoms^2^ |  |  | X |  |
| Enrollment |  | X |  |  |
| Height |  |  | X |  |
| Weight |  |  | X |  |
| Physical Exam, including vital signs and clinical signs of autoimmunity^3^ |  |  | X |  |
| Review of Concomitant Medications^4^ |  |  | X |  |
| ECOG status |  |  | X |  |
| ECG |  |  | X |  |
| Pregnancy Test (serum or urine) for women of childbearing potential (WOCBP)^5^ | X |  |  |  |
| Hematology^6^ |  |  | X |  |
| Serum Chemistry^7^ |  |  | X |  |
| Total IgG |  |  | X |  |
| Microscopic Urinalysis^8^ |  |  | X |  |
| Thyroid Function^9^ |  |  | X |  |
| HIV, Hepatitis screening^10^ |  |  | X |  |
| HLA Typing |  |  | X |  |
| Adverse Events |  |  | X |  |
| Tumor Imaging (CT, MRI)^11^ |  |  |  | X |

**1.** Includes review of systems, oncologic history and general medical history of all disease processes (active or resolved), and concomitant illnesses. Disease characteristics include diagnosis, date of diagnosis, cytology or histology, stage at time of trial entry, and breast cancer treatment history.

**2.** The Investigator should describe any signs and symptoms at presentation reported by patients during the 14 days prior to the initial dose of CP-675,206. Patients should be questioned about visual symptoms. Any patient who is experiencing symptoms suggestive of uveitis or melanoma-associated retinopathy (eg eye pain or redness, sensitivity to light or glare, blurred vision, floaters, night blindness, or visual field defects) should be evaluated by an ophthalmologist to rule out these conditions.

**3.** The physical exam should be performed by an Investigator at baseline, and a physician or designee may perform subsequent assessments, as appropriate. Vital signs include temperature, blood pressure, heart rate while sitting

**5.** Must be negative within 72 hours prior to the initial dose of CP-675,206.

**4.** All concomitant medications taken within 14 days need to be documented and recorded (including start and stop dates, as appropriate) on the patient CRF.

**6.** Hemoglobin, WBC with 5-part differential (percent and absolute values), platelet count.

**7.** Calcium, chloride, total protein, potassium, random glucose, sodium, blood urea nitrogen/BUN, creatinine, AST/SGOT, ALT/SGPT, alkaline phosphatase/ALP, lactic acid dehydrogenase/LDH, amylase, lipase, albumin, total bilirubin

**8.** Collect urine for Urinalysis [must include: 1) microscopic analysis of urinary sediment and 2) analysis for protein, glucose, and blood (other, as indicated)].

**9.** Thyroid stimulating hormone (TSH), T3, T4.

**10.** Refer to Exclusion Criteria.

**11.** Documentation of Baseline target and non target lesions by imaging techniques or by measurement of clinical lesion(s) must be performed within 28 days prior to dosing. The method of tumor assessment (CT, MRI) and tumor sites are to be consistent from baseline to the end of the study.

## **Table 2. On-trial assessments**

**Test/Assessment: Study Day Number:**

|  | D1 | D2 | D3 | D4 | D5 | D8  (1w) | D15  (2w) | D22  (3w) | D29  (4w) | D43  (6w) | D57 (8w) | D71  (10w) | D85  (12w) |
| --- | --- | --- | --- | --- | --- | --- | --- | --- | --- | --- | --- | --- | --- |
| Weight |  |  | X |  |  | X | X | X | X | X | X | X | X |
| Physical Exam, including signs of autoimmunity |  |  | X^1^ |  |  | X | X | X | X | X | X | X | X |
| ECOG status |  |  | X^1^ |  |  | X | X | X | X | X | X | X | X |
| Hematology^2,3,4^ | X |  |  |  |  | X | X | X | X | X | X | X | X |
| Serum Chemistry^3,4,5^ | X |  |  |  |  | X | X | X | X | X | X | X | X |
| Microscopic or Dipstick Urinalysis^3,4,6^ | X |  |  |  |  | X | X | X | X | X | X | X | X |
| Thyroid Function^3,4,7^ |  |  |  |  |  |  |  |  | X |  |  |  | X |
| Autoantibody Panel^3,4,8^ | X |  |  |  |  |  |  |  | X |  |  |  | X |
| Human anti-human antibody (HAHA)^9^ | X |  |  |  |  |  |  |  | X |  |  |  | X |
| Radiation^10^ | X | X | X | X | X |  |  |  |  |  |  |  |  |
| CP-675,206 Administration |  |  | X |  |  |  |  |  |  |  |  |  |  |
| Immune Monitoring^11^ | X |  |  |  |  | X | X |  | X |  | X |  | X |
| Blood specimen for genomic analysis^12^ | X |  |  |  |  |  |  |  |  |  |  |  |  |
| AE Monitoring^13^ | X | X | X | X | X | X | X | X | X | X | X | X | X |
| Tumor Imaging (CT, MRI)^14^ |  |  |  |  |  |  |  |  |  |  | X |  |  |

**1.** Tests performed/specimens drawn prior to dosing with CP-675,206.

**2.** Hemoglobin, WBC with 5-part differential (percent and absolute values), platelet count

**3.** Clinically significant abnormalities should be reported as Adverse Events in the CRF.

**4.** Assessments may be performed within 24 hours of the designated timepoints.

**5.** Calcium, chloride, total protein, potassium, random glucose, sodium, blood urea nitrogen/BUN, creatinine, AST/SGOT, ALT/SGPT, alkaline phosphatase/ALP, lactic acid dehydrogenase/LDH, amylase, lipase, albumin, total bilirubin

**6.** Complete urinalysis (with microscopic analysis of centrifuged sediment for casts or other abnormalities); performed at baseline. In subsequent evaluations, dipstick urine testing is allowed in lieu of microscopic analysis, however, microscopic analysis must be performed if dipstick results are abnormal.

**7.** Thyroid stimulating hormone (TSH), T3, T4. Thyroid function tests are also done as part of the pre-trial assessments (within 14 days before treatment begins).

**8.** The autoantibody panel will include antinuclear antibodies (ANA), antineutrophil cytoplasmic antibodies (ANCA), anti-DNA antibody (SS and DS), Rh factor (RhF), antithyroglobulin, anti liver-kidney microsomal (LKM), anti-islet-cell, antibodies to Ro (SSA) and to La (SSB) and antiphospholipid and/or other autoantibody tests.

**9.** Human anti-human antibody may be done at the Investigator’s discretion.

**10.** A total of 2000cGy to be delivered in 5 daily fractions using 6-18 MV photons.

**11.** Immune monitoring will be done using peripheral blood obtained by phlebotomy. PBMC and serum will be isolated and cryopreserved.

**12.** DNA extraction and banking for genomic evaluation.

**13.** Adverse event assessment to be performed continuously throughout the trial. Treatment-related adverse events will be followed until they resolve to baseline or Grade 1 or until the treating physician determines that they are irreversible.

**14.** Patients with measurable disease will be assessed by imaging every 8 weeks until progression (range 6-8 weeks). If a response is seen at week 8, the imaging will be repeated 4 weeks later. The same method of assessment and the same technique should be used for repeated measurements.

# 14. REFERENCES

1. Peggs KS, Quezada SA, Korman AJ, Allison JP. Principles and use of anti-CTLA4 antibody in human cancer immunotherapy. Curr Opin Immunol. 2006; 18:206-13.

2. Baecher-Allan C, Anderson DE. Immune regulation in tumor-bearing hosts. Curr Opin Immunol. 2006; 18:214-9.

3. Liyanage UK, Moore TT, Joo HG, Tanaka Y, Herrmann V, Doherty G, Drebin JA, Strasberg SM, Eberlein TJ, Goedegebuure PS, Linehan DC. Prevalence of regulatory T cells is increased in peripheral blood and tumor microenvironment of patients with pancreas or breast adenocarcinoma. J Immunol. 2002; 169:2756-61.

4. Leach DR, Krummel MF, Allison JP. Enhancement of antitumor immunity by CTLA-4 blockade. Science. 1996; 271:1734-6.

5. Hodi FS, Mihm MC, Soiffer RJ, Haluska FG, Butler M, Seiden MV, Davis T, Henry-Spires R, MacRae S, Willman A, Padera R, Jaklitsch MT, Shankar S, et al. Biologic activity of cytotoxic T lymphocyte-associated antigen 4 antibody blockade in previously vaccinated metastatic melanoma and ovarian carcinoma patients. Proc Natl Acad Sci U S A. 2003; 100:4712-7.

6. Beck KE, Blansfield JA, Tran KQ, Feldman AL, Hughes MS, Royal RE, Kammula US, Topalian SL, Sherry RM, Kleiner D, Quezado M, Lowy I, Yellin M, et al. Enterocolitis in patients with cancer after antibody blockade of cytotoxic T-lymphocyte-associated antigen 4. J Clin Oncol. 2006; 24:2283-9.

7. Camacho LH, Ribas A, Glaspy JA. Proc Am Soc Clin Oncol. 2004. 22:Abs#2505 (Abstr.).

8. Ribas A, Bozon VA, Lopez-Berestein G. Proc Am Soc Clin Oncol. 2005. 23:Abs #7524 (Abstr.).

9. Ribas A, Camacho LH, Lopez-Berestein G, Pavlov D, Bulanhagui CA, Millham R, Comin-Anduix B, Reuben JM, Seja E, Parker CA, Sharma A, Glaspy JA, Gomez-Navarro J. Antitumor activity in melanoma and anti-self responses in a phase I trial with the anti-cytotoxic T lymphocyte-associated antigen 4 monoclonal antibody CP-675,206. J Clin Oncol. 2005; 23:8968-77.

10. Sheard MA, Vojtesek B, Janakova L, Kovarik J, Zaloudik J. Up-regulation of Fas (CD95) in human p53wild-type cancer cells treated with ionizing radiation. Int J Cancer. 1997; 73:757-62.

11. Vereecque R, Buffenoir G, Gonzalez R, Cambier N, Hetuin D, Bauters F, Fenaux P, Quesnel B. gamma-ray irradiation induces B7.1 expression in myeloid leukaemic cells. Br J Haematol. 2008; 108:825-31.

12. Garnett CT, Palena C, Chakraborty M, Tsang KY, Schlom J, Hodge JW. Sublethal irradiation of human tumor cells modulates phenotype resulting in enhanced killing by cytotoxic T lymphocytes. Cancer Res. 2004; 64:7985-94.

13. Gaugler MH, Squiban C, van der MA, Bertho JM, Vandamme M, Mouthon MA. Late and persistent up-regulation of intercellular adhesion molecule-1 (ICAM-1) expression by ionizing radiation in human endothelial cells in vitro. Int J Radiat Biol. 1997; 72:201-9.

14. Hallahan DE, Virudachalam S. Accumulation of P-selectin in the lumen of irradiated blood vessels. Radiat Res. 1999; 152:6-13.

15. Quarmby S, Hunter RD, Kumar S. Irradiation induced expression of CD31, ICAM-1 and VCAM-1 in human microvascular endothelial cells. Anticancer Res. 2000; 20:3375-81.

16. Ganss R, Ryschich E, Klar E, Arnold B, Hammerling GJ. Combination of T-cell therapy and trigger of inflammation induces remodeling of the vasculature and tumor eradication. Cancer Res. 2002; 62:1462-70.

17. Reits EA, Hodge JW, Herberts CA, Groothuis TA, Chakraborty M, Wansley EK, Camphausen K, Luiten RM, de Ru AH, Neijssen J, Griekspoor A, Mesman E, Verreck FA, et al. Radiation modulates the peptide repertoire, enhances MHC class I expression, and induces successful antitumor immunotherapy. J Exp Med. 2006; 203:1259-71.

18. Demaria S, Kawashima N, Yang AM, Devitt ML, Babb JS, Allison JP, Formenti SC. Immune-mediated inhibition of metastases after treatment with local radiation and CTLA-4 blockade in a mouse model of breast cancer. Clin Cancer Res. 2005; 11:728-34.

19. Chakravarty PK, Alfieri A, Thomas EK, Beri V, Tanaka KE, Vikram B, Guha C. Flt3-ligand administration after radiation therapy prolongs survival in a murine model of metastatic lung cancer. Cancer Res. 1999; 59:6028-32.

20. Demaria S, Ng B, Devitt ML, Babb JS, Kawashima N, Liebes L, Formenti SC. Ionizing radiation inhibition of distant untreated tumors (abscopal effect) is immune mediated. Int J Radiat Oncol Biol Phys. 2004; 58:862-70.

21. Lohr F, Hu K, Haroon Z, Samulski TV, Huang Q, Beaty J, Dewhirst MW, Li CY. Combination treatment of murine tumors by adenovirus-mediated local B7/IL12 immunotherapy and radiotherapy. Mol Ther. 2000; 2:195-203.

22. Oh YT, Chen DW, Dougherty GJ, McBride WH. Adenoviral interleukin-3 gene-radiation therapy for prostate cancer in mouse model. Int J Radiat Oncol Biol Phys. 2004; 59:579-83.

23. Nikitina EY, Gabrilovich DI. Combination of gamma-irradiation and dendritic cell administration induces a potent antitumor response in tumor-bearing mice: approach to treatment of advanced stage cancer. Int J Cancer. 2001; 94:825-33.

24. Kudo-Saito C, Schlom J, Camphausen K, Coleman CN, Hodge JW. The requirement of multimodal therapy (vaccine, local tumor radiation, and reduction of suppressor cells) to eliminate established tumors. Clin Cancer Res. 2005; 11:4533-44.

25. Linsley PS, Greene JL, Tan P, Bradshaw J, Ledbetter JA, Anasetti C, Damle NK. Coexpression and functional cooperation of CTLA-4 and CD28 on activated T lymphocytes. J Exp Med. 1992; 176:1595-604.

26. Lugade AA, Moran JP, Gerber SA, Rose RC, Frelinger JG, Lord EM. Local radiation therapy of B16 melanoma tumors increases the generation of tumor antigen-specific effector cells that traffic to the tumor. J Immunol. 2005; 174:7516-23.

27. Cleator S, Heller W, Coombes RC. Triple-negative breast cancer: therapeutic options. Lancet Oncol. 2005; 8:235-44.

28. Bauer KR, Brown M, Cress RD, Parise CA, Caggiano V. Descriptive analysis of estrogen receptor (ER)-negative, progesterone receptor (PR)-negative, and HER2-negative invasive breast cancer, the so-called triple-negative phenotype: a population-based study from the California cancer Registry. Cancer. 2007; 109:1721-8.

29. Perou CM, Sorlie T, Eisen MB, van de RM, Jeffrey SS, Rees CA, Pollack JR, Ross DT, Johnsen H, Akslen LA, Fluge O, Pergamenschikov A, Williams C, et al. Molecular portraits of human breast tumours. Nature. 2000; 406:747-52.

30. Bertucci F, Finetti P, Cervera N, Charafe-Jauffret E, Mamessier E, Adelaide J, Debono S, Houvenaeghel G, Maraninchi D, Viens P, Charpin C, Jacquemier J, Birnbaum D. Gene expression profiling shows medullary breast cancer is a subgroup of basal breast cancers. Cancer Res. 2006; 66:4636-44.

31. Yakirevich E, Izhak OB, Rennert G, Kovacs ZG, Resnick MB. Cytotoxic phenotype of tumor infiltrating lymphocytes in medullary carcinoma of the breast. Mod Pathol. 1999 ; 2:1050-6.

32. Tamiolakis D, Simopoulos C, Cheva A, Lambropoulou M, Kotini A, Jivannakis T, Papadopoulos N. Immunophenotypic profile of tumor infiltrating lymphocytes in medullary carcinoma of the breast. Eur J Gynaecol Oncol. 2002; 23:433-6.

33. Georgiannos SN, Renaut A, Goode AW, Sheaff M. The immunophenotype and activation status of the lymphocytic infiltrate in human breast cancers, the role of the major histocompatibility complex in cell-mediated immune mechanisms, and their association with prognostic indicators. Surgery. 2003; 134:827-34.

34. Kuroda H, Tamaru J, Sakamoto G, Ohnisi K, Itoyama S. Immunophenotype of lymphocytic infiltration in medullary carcinoma of the breast. Virchows Arch. 2005; 446:10-4.

**Attachment B**

**Budget**

###### Funding:

The total approved funding for the Study to be conducted by Sponsor-Investigator is TWO HUNDRED FOURTY SIX THOUSAND FIVE HUNDRED FOURTY FIVE CANADIAN DOLLARS ($246,545.00 CAD). Such amounts are inclusive of the Thirty (30%) institutional overhead and does not include any applicable taxes.

###### Product:

Drug Name and Strength and Quantity to be provided:

| Drug Name: | Tremelimumab |
| --- | --- |
| Strength: | 20mg/mL |
| Quantity: | 1000 vials |

Should additional supplies of study drug be required based on scientific justification, Sponsor-Investigator will request from Pfizer, in writing such revised quantities. Upon Pfizer’s receipt/review of request and at Pfizer’s sole discretion a decision will be rendered and communicated to the Sponsor-Investigator in writing *i.e. email/facsimile/confirmation letter.*

###### Funding Payment Schedule:

$46,545 Payable upon Pfizer’s receipt of fully executed Agreement and Health Canada approval (if applicable).

$75,000 Payable upon recruitment of 50% of projected amount of patients

$75,000 Payable upon 100% of recruitment

$50,000 Payable upon Pfizer’s receipt of results

###### **Payee Information:**

**Payee Name (as it will appear on the cheque):**

University Health Network – Attn: Dr. Srikala Sridhar

610 University Avenue

Room 5-222

Toronto, ON M5G 2M9

**ATTACHMENT C**

**PFIZER ANTI-BRIBERY AND ANTI-CORRUPTION PRINCIPLES**

Pfizer’s corporate policies provide that Pfizer colleagues must conduct all Pfizer business in a lawful and ethical manner, in accordance with applicable laws and regulations, including the U.S. Foreign Corrupt Practices Act of 1977 (the “**FCPA**”). The FCPA prohibits making, promising, or authorizing the making of a corrupt payment or providing anything of value to a Government Official to induce that official to make any governmental act or decision to assist a company in obtaining or retaining business. The FCPA also prohibits a company or person from using another company or individual to engage in any of the foregoing activities. As a U.S. company, Pfizer must comply with the FCPA and, as such, requires that its consultants, agents, representatives, and a companies acting on its behalf (“**Business Associates**”) do the same. Consequently, Pfizer requires all of its Business Associates to conduct themselves in accordance with these principles.

***Definition of a Government Official***

Government Official:

(a) an officer or employee of, or (b) an individual who acts in an official capacity on behalf of:

(i) an entity listed in Schedule FCPA1 (see next page),

(ii) any other Canadian federal, provincial, territorial or municipal government, agency, organization, commission, committee or foundation, or

(iii) a Canadian political party, Canadian Crown corporation or public international organization or

(c) a Canadian political party itself.

Note, despite the fact that Canada operates under a public health care system, physicians are generally independent contractors and are not generally considered employees of the government.

The term “**Government**” is meant to include all levels and subdivisions of government (i.e., local, regional or national and administrative, legislative or executive). Because this definition of “government official” is so broad, it is likely that Business Associates will interact with a government official in the ordinary course of their business.

***FCPA, Anti-Corruption and Anti-Bribery Principles***

Business Associates may not directly or indirectly make, promise, or authorize the making of a corrupt payment or provide anything of value to any Government Official to induce that Government Official to make any governmental act or decision to help Pfizer obtain or retain business. Business Associates may never make a payment to or offer a Government Official any item or benefit, regardless of value, as an improper inducement for such Government Official to approve, reimburse, prescribe, or purchase a Pfizer product, to influence the outcome of a clinical trial, or otherwise improperly to benefit Pfizer’s business activities.

***Understand and Follow Local Laws***

Business Associates need to understand whether local laws, regulations, or operating procedures (including requirements imposed by government entities such as state-owned hospitals or research institutions) impose any limits, restrictions, or disclosure requirements on compensation, financial support, donations, or gifts that may be provided to Government Officials. Business Associates must take into account and comply with any applicable restrictions in conducting their Pfizer-related activities. If a Business Associate is uncertain as to the meaning or applicability of any identified limits, restrictions, or disclosure requirements with respect to interactions with Government Officials, that Business Associate should consult with his or her primary Pfizer contact before undertaking their activities.

ATTACHMENT “C” (cont.)

Schedule FCPA1

GOVERNMENT BODIES WITH WHICH PFIZER COMMONLY INTERACTS

| - Health Canada - Industry Canada - Privy Council Office - Prime Minister's Office - Foreign Affairs and International Trade - Patented Medicine Prices Review Board - Veterans Affairs Canada - National Defence - Finance Canada - Royal Canadian Mounted Police - Common Drug Review (CDR) | - Canadian Agency for Drugs and Technologies in Health (CADTH) - Integrated Health Agencies (Atlantic Canada) - Réseaux locaux de services de santé et de services sociaux (CSSS - successor to CLSCs) (Québec) - Groupes de médecine familiale (Québec) - Cliniques réseau (CR or CMA) (Québec) - Local Health Integration Network (Ontario) - Family Health Teams (Ontario) - Regional Health Authorities (Western Canada) |
| --- | --- |

EXAMPLES OF GOVERNMENT OFFICIALS INCLUDE:

- Elected or appointed government officials;
- Public servants;
- Declared political candidates (whether for party nomination or election);
- HCPs who meet the criteria set forth in the definition of Government Official, *e.g.*, HCPs employed by (a) the military, (b) the Correctional Service of Canada (prisons and penitentiaries) or (c) government operated or controlled hospitals or institutions (*e.g.*, mental institutions, veterans affairs hospitals) and HCPs serving on government task forces or committees (*e.g.*, Management of Severe Pain Advisory Committee of Experts, AIDS Advisory Committee, National Advisory Council on Aging, Medical Advisors Group);
- Officers, employees or individuals who act in an official capacity on behalf of the United Nations, World Health Organization, World Trade Organization, International Joint Commission – United States and Canada, International Committee of the Red Cross, North American Development Bank, International Monetary Fund, International Criminal Police Organization (INTERPOL) and Inter-American Development Bank; and

Officers, employees or individuals who act in an official capacity on behalf of the School Boards & Community Colleges.

ATTACHMENT “D”

**COMPLIANCE CERTIFICATION**

Pursuant to this Agreement between Pfizer Canada Inc. and the undersigned, dated __________________, the undersigned hereby certifies:

it has been provided with a copy of the *Pfizer Anti- Bribery and Anti-Corruption Principles*; and it has not made any payments or provided any benefit to a Government Official, as defined in the *Pfizer Anti-Bribery and Anti-Corruption Principles*, to induce such Government Official to make any governmental act or decision to help Pfizer obtain or retain business and have not made a payment or offered any item or benefit, regardless of value, as an improper inducement for such Government Official to approve, reimburse, prescribe, or purchase a Pfizer product, to influence the outcome of a clinical trial, or otherwise improperly to benefit Pfizer’s business activities.

| INSTITUTION: |  |
| --- | --- |
| SIGNATURE: |  |
| NAME: |  |
| TITLE: |  |
| DATE: |  |

| SPONSOR-INVESTIGATOR: |  |
| --- | --- |
| SIGNATURE: |  |
| NAME: |  |
| TITLE: |  |
| DATE: |  |
